# Using Whole Genome Sequences to Investigate Adenovirus Outbreaks in a Hematopoietic Stem Cell Transplant Unit

**DOI:** 10.3389/fmicb.2021.667790

**Published:** 2021-07-02

**Authors:** Chloe E. Myers, Charlotte J. Houldcroft, Sunando Roy, Ben K. Margetts, Timothy Best, Cristina Venturini, Jose A. Guerra-Assunção, Charlotte A. Williams, Rachel Williams, Helen Dunn, John C. Hartley, Kanchan Rao, Kathryn J. Rolfe, Judith Breuer

**Affiliations:** ^1^Cambridge Clinical Microbiology and Public Health Laboratory, Public Health England, Cambridge, United Kingdom; ^2^Department of Medicine, University of Cambridge, Cambridge, United Kingdom; ^3^Division of Infection and Immunity, University College London, London, United Kingdom; ^4^Division of Infection, Immunity and Inflammation, Great Ormond Street Institute of Child Health, University College London, London, United Kingdom; ^5^Department of Microbiology, Virology and Infection Prevention and Control, Great Ormond Street Hospital for Children National Health Service Foundation Trust, London, United Kingdom

**Keywords:** adenovirus, epidemiology, whole genome sequencing, pediatric infectious disease, nosocomial transmission

## Abstract

A recent surge in human mastadenovirus (HAdV) cases, including five deaths, amongst a haematopoietic stem cell transplant population led us to use whole genome sequencing (WGS) to investigate. We compared sequences from 37 patients collected over a 20-month period with sequences from GenBank and our own database of HAdVs. Maximum likelihood trees and pairwise differences were used to evaluate genotypic relationships, paired with the epidemiological data from routine infection prevention and control (IPC) records and hospital activity data. During this time period, two formal outbreaks had been declared by IPC, while WGS detected nine monophyletic clusters, seven were corroborated by epidemiological evidence and by comparison of single-nucleotide polymorphisms. One of the formal outbreaks was confirmed, and the other was not. Of the five HAdV-associated deaths, three were unlinked and the remaining two considered the source of transmission. Mixed infection was frequent (10%), providing a sentinel source of recombination and superinfection. Immunosuppressed patients harboring a high rate of HAdV positivity require comprehensive surveillance. As a consequence of these findings, HAdV WGS is being incorporated routinely into clinical practice to influence IPC policy contemporaneously.

## Introduction

Clinical infections caused by human mastadenoviruses (HAdVs) are associated with significant morbidity (10–89%) and mortality (6–70%) in the immunocompromised host (Echavarría, 2008). Risk factors for poor outcome include pediatric patients (who are susceptible to primary infection), unrelated donor stem cell transplants (SCTs), graft-vs.-host disease, T-cell depletion of graft, and certain immunosuppressive drug regimens (Shields et al., 1985; Runde et al., 2001; Chakrabarti et al., 2004).

The burden of HAdV infection is significant; within the pediatric oncology population, HAdV has been reported to account for 15% of all diarrhoeal cases (Mhaissen et al., 2017). Amongst pediatric patients undergoing hematopoietic stem cell transplant (HSCT), HAdV viremia and stool shedding were found in 15 and 42% of patients, respectively (Hiwarkar et al., 2013; Kosulin et al., 2018). As non-enveloped viruses, HAdVs can be resistant to standard alcohol cleaning regimens and can survive as clinically infectious particles for up to 4 weeks (Gordon et al., 1993). Nosocomial transmission has been frequently reported in the literature (Russell et al., 2006; Rutala et al., 2006); however, the identification of these outbreaks is likely to be under-reported due to the limitations of existing HAdV typing protocols that are performed infrequently and target only small regions of selected genes (Seto et al., 2013).

Advances in whole genome sequencing (WGS) have provided valuable insights into the molecular epidemiology of a number of key hospital pathogens (Brodrick et al., 2016; Eyre et al., 2017; Brown et al., 2019; Roy et al., 2019). This has been well-illustrated recently in the context of severe acute respiratory syndrome coronavirus 2 (SARS-CoV-2), where the application in real time has allowed prompt feedback supporting epidemiological links and the utility of the existing IPC policies (Meredith et al., 2020).

Specifically, within our population, a tertiary pediatric referral center in which 30% of patients are immunocompromised, HAdV is one of the leading causes of viral gastroenteritis, comprising 44% of all infections (Brown et al., 2016). Over the last financial year (2019–2020), there were 642 new HAdV detections, from any sample site, 99 of which were viremias (local audit data; all patient groups). Adenoviremia significantly decreases the probability of survival in children following HSCT and also increases the duration of inpatient hospital stay with an associated financial burden (Faden et al., 2005; Mattner et al., 2008; Hiwarkar et al., 2013; Swartling et al., 2015).

There is a wide range of adenovirus infections: primary new infections with a second strain, reactivation of a previously known infection, reactivation of a previously unknown quiescent infection, or a mixture of these. Due to these potential overlapping scenarios, the routine epidemiological data can only suggest that a specific infection may be healthcare, as opposed to community, associated but cannot confirm or refute it. Adenovirus typing to species or serotype level may refute a cross-infection hypothesis, but it does not provide adequate discrimination to confirm cross infection, for which WGS is necessary (Houldcroft et al., 2018).

Extensive efforts are employed to prevent adenovirus infection, modified to account for local suspected transmission routes. In our unit, we have implemented rigorous IPC policies including environmental screening (Pankhurst et al., 2014; Cloutman-Green et al., 2015), but transmission is still suspected. In our hospital, over a 20-month period, seven HAdV outbreaks have been investigated by the IPC team, two of them were associated with the HSCT unit, and there had been five adenovirus-associated deaths.

To further understand the routes of transmission and enable further development of the infection control policy, we undertook extensive epidemiological investigation and sequencing of isolates from the HSCT unit to determine what proportion was transmitted. Using the WGS data, we documented the genetic relatedness between isolates and described the possible transmission events. These findings can be used to interrupt HAdV transmission dynamics and should be used to further develop routine IPC policy and ultimately improved patient care.

## Materials and Methods

### Context and Ethics

Great Ormond Street Hospital (GOSH) is a 350-bed, pediatric tertiary referral center. Due to the immunocompromised status of patients referred here, over 60% of beds are single room isolation facilities. In addition to those patients who are symptomatic, “high-risk” patients—those who are admitted for hematological transplant or congenital immunodeficiencies—are screened weekly and on admission for gastrointestinal infection using polymerase chain reaction (PCR). The PCR methods used by the GOSH diagnostic laboratory have been described previously (Houldcroft et al., 2018). Residual diagnostic samples were collected from patients with PCR confirmed HAdV infection. The PCR cycle threshold (C_*T*_) values provided a comparable semiquantitative indicator of viral titer. The use of these samples for research was approved by The National Research Ethics Service Committee London—Fulham (reference: 17/LO/1530). The clinical data were extracted from the hospital databases by the GOSH Digital Research Environment (DRE) team and linked to an anonymized patient number.

### Definitions, Patients, and Samples

A HSCT unit nosocomial outbreak is suspected when any new detection of HAdV infection is identified in a child who was negative on admission screening. Further information on the routine management of outbreaks is provided in Supplementary Material. For surveillance reporting, healthcare acquired infection (HCAI) is defined as a positive diagnostic sample ≥48 h post-admission and community-acquired infection (CAI) defined as a positive diagnostic sample within 48 h of admission and no healthcare contact in the preceding 14 days.

A total of 169 samples from 74 patients were included in this study (Supplementary Table 1). All patients were known to have either a congenital or acquired immunodeficiency, and therefore considered high risk. As part of this investigation, 11 outbreak samples (*n* = 8 patients) were identified as two clusters by IPC [infection control cluster one (ICC 1) patients: 54, 55, 56, 57, 62, and 68, and infection control cluster two (ICC 2) patients: 40 and 38] and 37 non-outbreak samples (*n* = 29 patients, including HCAI and CAI infections) were sequenced and analyzed with a local database of HAdV sequences (127 sequences from 37 patients).

### SureSelect Bait Design and Sequencing

Methods allowing high-throughput HAdV WGS directly from clinical samples have been developed (Depledge et al., 2011; Batty et al., 2013; Houldcroft et al., 2018). These methods provide a proof of concept that WGS offers the resolution required to confirm nosocomial transmission of HAdV; however, there were technical improvements to be made with species C viruses (85/107 clinical samples) yielding lower quality sequences (Houldcroft et al., 2018). 120-mer baits (version 2) were redesigned, using an in-house Perl script with a tiling factor of 12 × (each position in a given genome is covered by 12 unique bait designs) against all whole HAdV sequences (487) in GenBank (accessed on 24 January 2018). The bait design was uploaded to SureDesign, and biotinylated RNA oligonucleotides (baits) were synthesized by Agilent Technologies, Santa Clara, California (Agilent Technologies, 2021).

Quality control of sample DNA, library preparation using the SureSelect^XT^ Illumina paired-end protocol, and sequencing on an Illumina MiSeq sequencer were performed as described earlier (Houldcroft et al., 2018), except the utilization of the SureSelect^XT^ low input kit. Base calling and sample demultiplexing were performed as standard for the MiSeq platform, generating paired FASTQ files for each sample.

### Genome Mapping, Assembly, and Phylogenetic Analysis

Sequences for all 169 samples were assembled using a reference-based pipeline in CLC Genomics Workbench version 12.0.1 (QIAGEN, Hilden, Germany); the detailed methodology can be found in Supplementary Figure 1. Briefly, all reads were quality trimmed and the adaptor sequences were removed. The trimmed reads were mapped to a reference database (*n* = 103), where 90% of each read mapped with a minimum of 90% identity, the best reference match was used to assign a genotype to each sample. If mapped reads generated a good match to more than one genotype, suggesting a mixed infection, samples underwent further investigation (Supplementary Figures 1–5 and Supplementary Tables 2–6).

Once a sample had been assigned a genotype, a second pipeline was then used to quality trim, re-map to the best reference match with a length and similarity fraction of 0.8, before extracting a consensus sequence. The areas of low coverage (<10-fold) were assigned the ambiguity symbol N.

Robust consensus sequences are required for the downstream analysis; therefore, only samples achieving ≥90% genome coverage and ≥100-fold average read depth (quality cut-off) were included in further analysis. Consensus sequences (GenBank accessions MW686757–MW686857) were aligned, and phylogenies were constructed using CLC Genomics Workbench (version 12.0.1) (Supplementary Material). Pairwise single-nucleotide variant counts were computed using Molecular Evolutions Genetics Analysis (MEGA) software version 6 (Kumar et al., 2016).

### Epidemiological Support of Phylogenetic Clusters

Where there were sufficient HAdV sequences to clearly distinguish local UK variants from non-UK reference sequences (species A, C, and F), monophyletic clusters, defined as groups comprising two or more samples from at least two patients arising from a common ancestral node, with bootstrap support ≥90% were used to identify the putative outbreaks. Where there were insufficient sequences to provide adequate phylogenetic resolution (species B HAdVs), two or more samples within a monophyletic cluster with short branch lengths (<0.002) were used. Timelines for each monophyletic cluster were visualized using the ggplot2 library (Wickham, 2016), incorporating patient admission data and HAdV PCR positivity. Patients within a cluster were defined as epidemiologically supported if they were present on the same ward or unit becoming positive during the incubation period of the virus [median 5.6 days (95% CI 4.8–6.3) based on respiratory disease (Lessler et al., 2009)] and unsupported if they became positive during admissions to completely different wards or had no links with any other sequenced patient.

### Statistical Analysis

Statistical tests were performed using two-tailed tests at the 5% significance level within GraphPad Prism version 8.3.0 for Mac OS, GraphPad Software, San Diego, CA, United States, www.graphpad.com (Supplementary Material).

## Results

### Burden of Infection and Viral Genotypes

**Figure 1:**
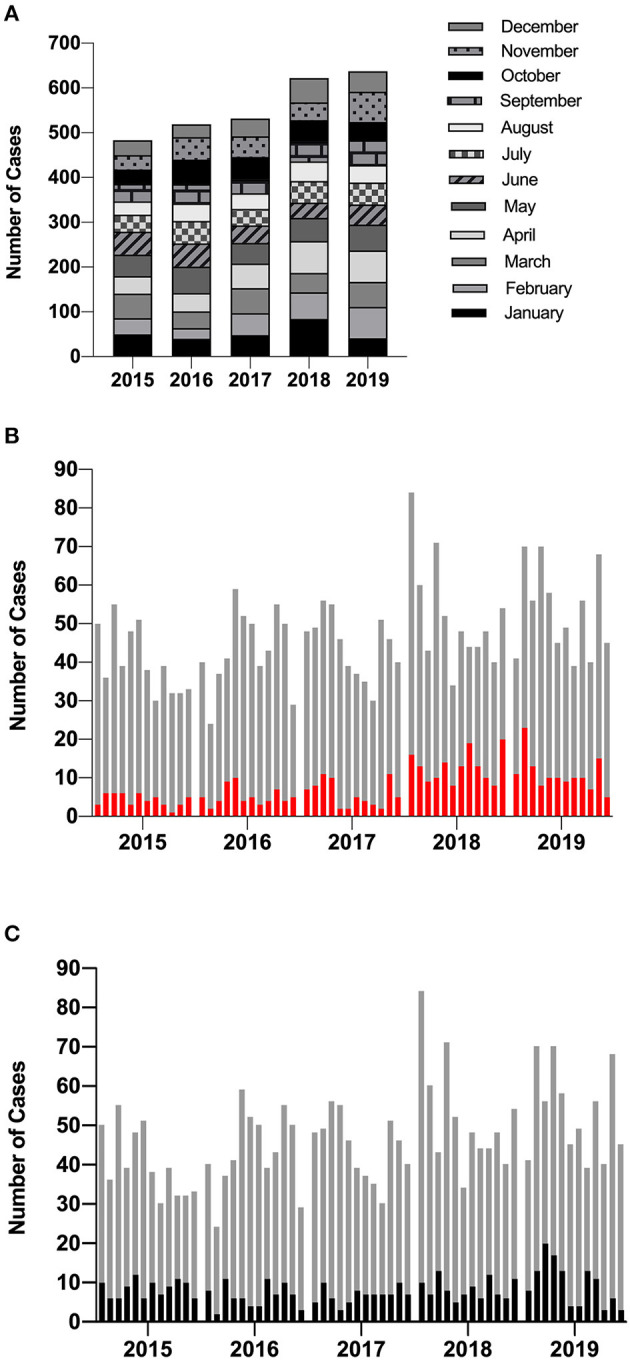
Incidence of new HAdV cases diagnosed using PCR by the diagnostic laboratory. **(A)** The total number of HAdV cases identified by GOSH increased each year but did not demonstrate any seasonality. **(B)** The proportion of new positives that were documented as HCAI are shown in red and **(C)** the proportion of new positives identified from patients admitted to the high-risk HSCT unit are highlighted in black. Each column represents a calendar month. HAdV, human mastadenovirus; PCR, polymerase chain reaction; GOSH, Great Ormond Street Hospital; HCAI, healthcare-acquired infection.

Routine reporting of the first HAdV PCR-positive cases by the diagnostic laboratory between 2015 and 2019 is summarized in Figure 1. As expected, no seasonality was observed (Brown et al., 2016); however, cases increased each year with a marked rise between 2017 and 2018 (Figure 1A). The proportion of new cases that were attributed to HCAI during this time period rose from 12% (2015–2017) to 23% (2018–2019) (Figure 1B). On average, 18% of new positives are detected from patients admitted to the high-risk HSCT unit (Figure 1C).

Between August 2017 and April 2019, IPC identified 11 outbreak samples (from ICC 1 and ICC 2) and 37 non-outbreak samples (including HCAI and CAI cases) from high-risk patients. Sequences were analyzed with 121 previously sequenced samples. A total of 169 clinical samples containing HAdV genotypes A31 (14%), B3 (2%), B11 (1%), C1 (17%), C2 (21%), C5 (18%), C89 (8%), E4 (1%), and F41 (3%) from 74 patients with either localized (e.g., eye, respiratory, and digestive) or disseminated infection were included. Seven of these samples (4%) failed to sequence and 17 (10% of patients) had mixed HAdV infections (Supplementary Table 1 and Supplementary Figure 6).

### Improved Sequencing Quality

Of 169 samples across all genotypes, 56 (42%) of 132 HAdV genomes passed the quality cut-off using version 1 baits and 46 (85%) of 54 HAdV genomes passed using version 2 baits (Supplementary Table 1 and Supplementary Figure 7). Both genome coverage and on-target reads were statistically significantly improved for species C viruses using version 2 baits (Supplementary Figure 8). This was despite similar species C viral titers in samples between bait groups (Supplementary Figure 9). Average read depth improved but remained significantly lower for species C viruses regardless of the baits used (*P* = 0.0002 version 1 baits vs. *P* = 0.05 version 2 baits).

Out of the seven samples that failed to sequence, four were sequenced using version 1 baits. Three samples failed using version 2 baits, one with a viral load that had previously been successful. There was an insufficient sample for repeat testing. Using an estimated linear regression model, it is predicted that samples with HAdV C_T_ values of ≤ 34 would generate a ≤ 100-fold read depth, with 95% certainty (Supplementary Figure 10).

### Phylogenetic Investigation of Outbreaks and Deaths

**Figure 2:**
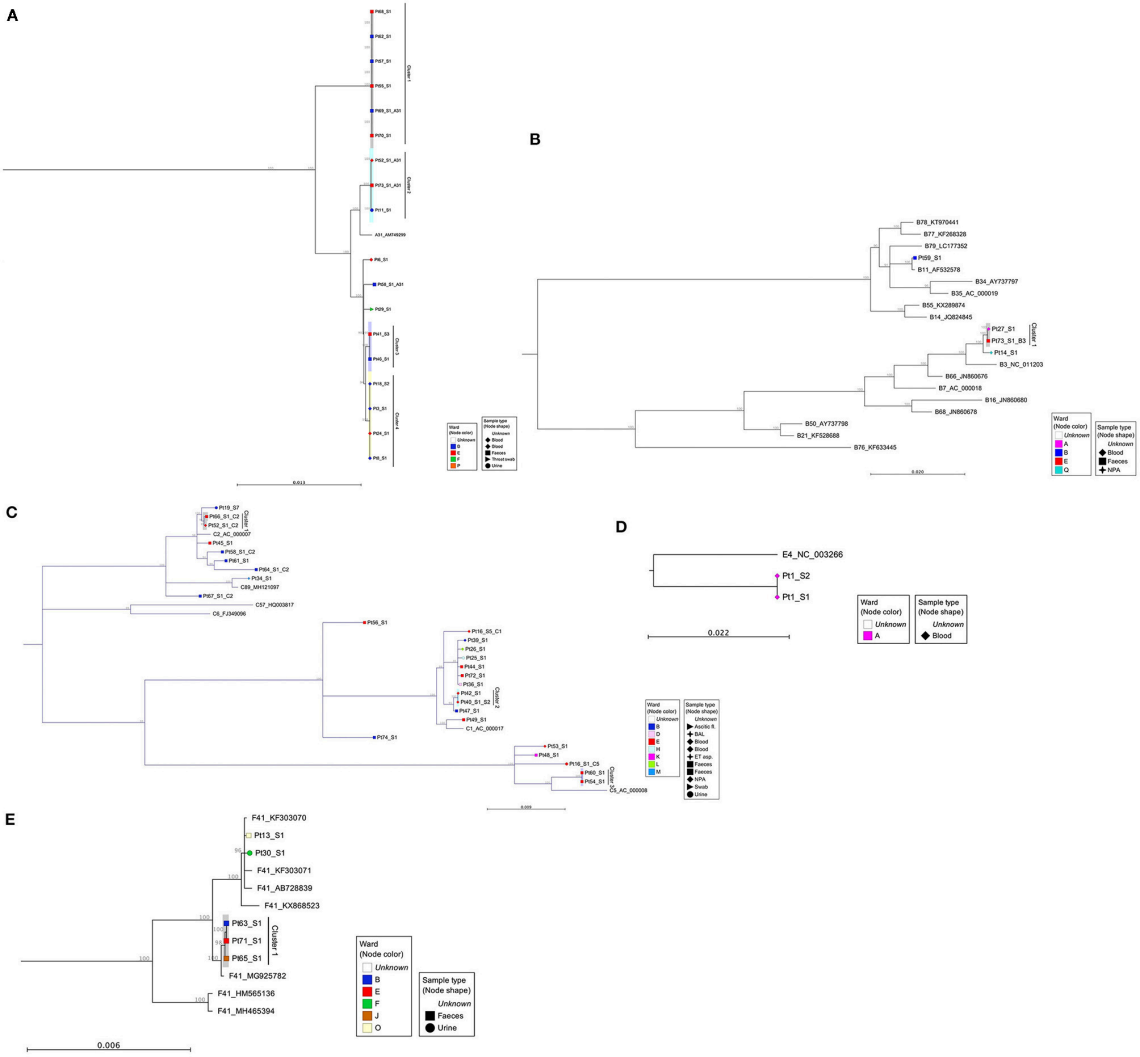
Maximum likelihood phylogenies of adenovirus full genome sequences, organized by species: **(A)** species A viruses, **(B)** species B viruses, **(C)** species C viruses, **(D)** species E, and **(E)** species F viruses. Sequences were aligned and maximum likelihood phylogenies generated using CLC Genomics Workbench (version 12.0.1), 500 bootstraps. Clinical samples are labeled according to their anonymized patient number (PtX) and specimen number (_SX). Additional samples from sequentially sampled patients have been collapsed. Inter-species reference sequences (A12, A18, and A61) were also removed from phylogeny A and from phylogeny E (F40) to aid visualization of patient samples. The shape of each node correlates with the sample type and color, the ward on which the sample was taken. Reference sequences are identified by their HAdV genotype and GenBank Accession. A bootstrap threshold of 80% is shown. Any clinical samples from at least two patients, with a bootstrap support >90, were considered a cluster. The parsimony informative site for each phylogeny is as follows: (A) 2979/34444, (B) 7054/36263, (C) 3104/36332, (D) 1348/36046, and (E) 4455/34469. For phylogeny D, there are only three sequences, out of which two are identical. In this case, the sites reported are only the variable sites between the two groups. NPA, nasopharyngeal aspirate; BAL, bronchoalveolar lavage; ET aspirate, endotracheal aspirate.

To substantiate nosocomial transmission, maximum likelihood phylogenies were constructed (Figure 2). Nine monophyletic clusters were identified (Figures 2A–E) and summarized in Table 1. One of these clusters (A31 Cluster 4) had been previously identified phylogenetically (Houldcroft et al., 2018).

**Table 1:** Summary of monophyletic clusters identified by maximum likelihood phylogeny using whole HAdV genome sequences.

**HAdV type, sequence cluster number**	**Sample code**	**ICC number**	**IPC record**	**Ward involved**	**Temporally related^a^**	**Diversity within cluster^b^**	**Conclusion**
A31 Cluster 1	Pt69_S1_A31	–	HCAI not linked to outbreak	B	Yes	0	Confirmed transmission cluster
	Pt70_S1	–	HCAI not linked to outbreak	E	Yes		
	Pt68_S1	1	Chronic HAdV–ICC 1 investigated	E	Yes		
	Pt62_S1	1	HCAI–ICC 1 investigated	B	Yes		
	Pt57_S1	1	HCAI–ICC 1 investigated	B	Yes		
	Pt55_S1	1	HCAI–ICC 1 investigated	E	Yes		
A31 Cluster 2	Pt11_S1	–	Not classified	B	No	6	Likely transmission, unconfirmed
	Pt73_S1_A31	–	HCAI	E	Yes	3	Confirmed transmission cluster
	Pt52_S1_A31^*^	–	HCAI	E	Yes		
A31 Cluster 3	Pt41_S1	–	CAI	E	Yes	1	Confirmed transmission cluster
	Pt46_S1	–	CAI	B	Yes		
A31 Cluster 4	Pt24_S1	–	Not classified	E	Yes	0–1	Confirmed transmission cluster
	Pt8_S1	–	Not classified	B	No		
	Pt18_S1	–	Probable HCAI	B	Yes		
	Pt3_S1	–	Not classified	B	Yes		
B3 Cluster 1	Pt27_S1	–	Not classified	A	No	13	Unlikely transmission cluster
	Pt73_S1_B3		Marked as long-term carriage from previous admission	E	No		
C2 Cluster 1	Pt52_S1_C2^*^	–	HCAI	E	No	6	Likely transmission, unconfirmed
	Pt66_S1_C2	–	HCAI	E	No		
C1 Cluster 2	Pt42_S1	–	CAI	E	Yes	0	Confirmed transmission cluster
	Pt40_S1_S2	2	HCAI–ICC 2 investigated	E	Yes		
C5 Cluster 3	Pt60_S1^*^	–	Not classified	E	Yes	0	Confirmed transmission cluster
	Pt54_S1	1	HCAI–ICC 1 investigated	E	Yes		
F41 Cluster 1	Pt71_S1	–	HCAI	E	Yes	0	Confirmed transmission cluster
	Pt63_S1	–	HCAI	B	Yes		
	Pt65_S1	–	CAI	B	Yes	3	Confirmed transmission cluster

*HAdV, human mastadenovirus; ICC, Infection Control Cluster; IPC, Infection Prevention and Control; HCAI, healthcare-acquired infection; CAI, community acquired infection*.

a *Temporally related, HAdV PCR positive whilst admitted to same/linked ward*.

b *Diversity within cluster, expressed as the number of pairwise differences/single-nucleotide polymorphisms (SNPs) across the whole genome*.

* *Indicates patients who died from or in association with overwhelming HAdV infection*.

Four of the six patients from ICC 1 (patients 55, 57, 62, and 68) were phylogenetically linked (Figure 2A, A31 Cluster 1). Additionally, two patients (Pt69 and Pt70) documented as having HCAI, for whom no source of infection had previously been identified were linked phylogenetically to ICC 1. One patient (Pt69) involved in monophyletic A31 Cluster 1 had a mixed HAdV infection. The sequence data obtained from Pt38 were of insufficient quality to confirm or refute transmission with Pt40 of ICC 2 phylogenetically; however, one of these patients, Pt40, was phylogenetically linked to another patient, Pt42, who had a concurrent HAdV-C2 infection. WGS identified an additional six monophyletic clusters, involving 16 patients who had not previously been identified by standard IPC follow-up (Figure 2 and Table 1).

Out of five patients who died (patients 52, 53, 59, 60, and 61) as a result of or in association with overwhelming HAdV infection (Supplementary Table 1), two were linked to a monophyletic cluster. Patient 52 was found to have a mixed genotype (C2 and A31) infection that was dominated by a phylogenetically unlinked C2 (Figure 2C) but with a minority subpopulation of A31 that clustered with two other patients (11 and 73, A31 Cluster 2, Figure 2A). Patient 60 had a single C5 infection that clustered with Pt54 (C5 Cluster 3, Figure 2C). The remaining three patients had phylogenetically unlinked single genotype infections.

### Traditional Epidemiology, Contact Tracing Supported Phylogeny Assignments

**Figure 3:**
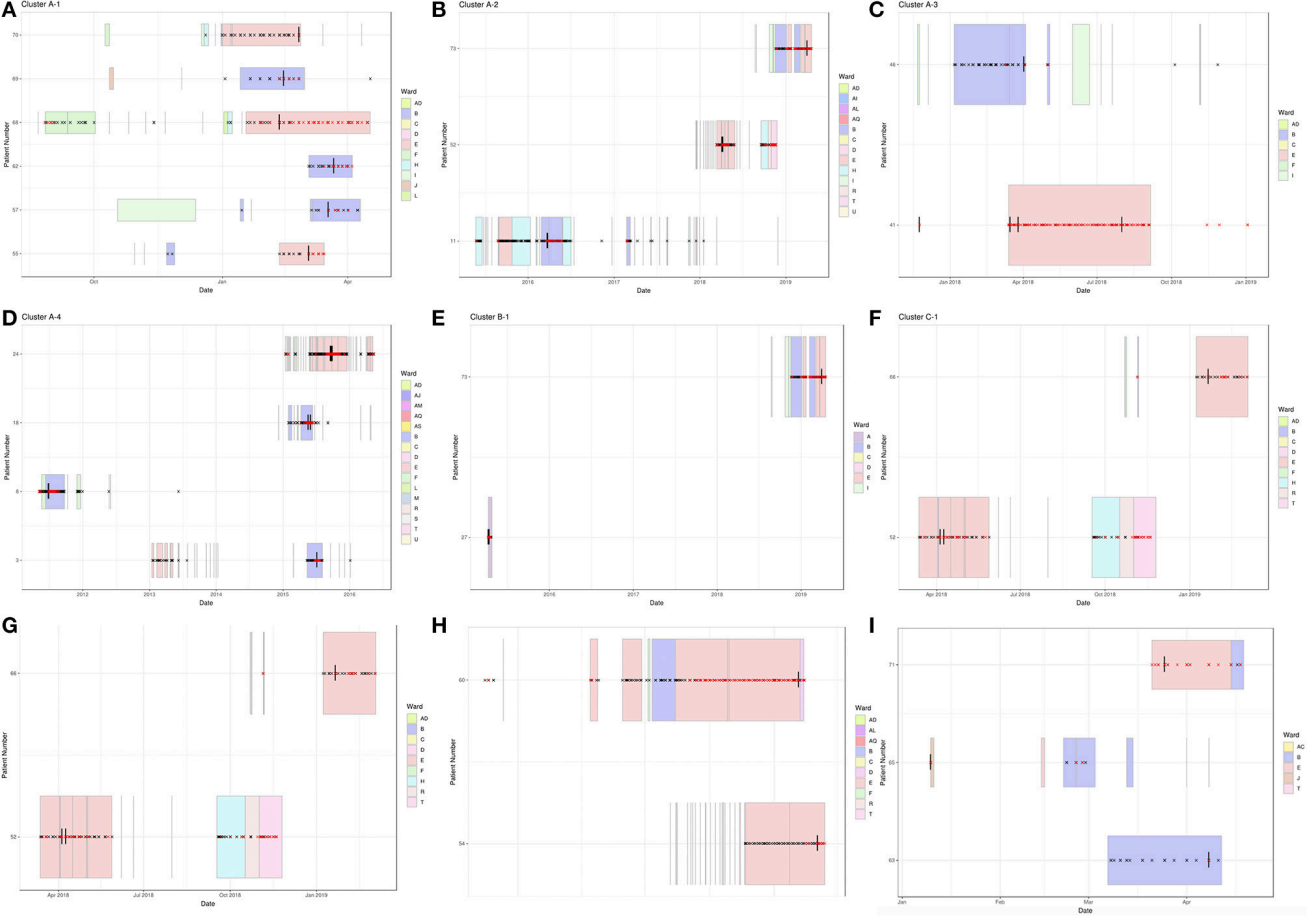
Temporal relationship between HAdV patients forming monophyletic clusters. Clinical data were extracted from hospital databases by the GOSH Digital Research Environment (DRE) team and linked to an anonymized patient number. The ggplot2 library was used to visualize the data between positive adenovirus PCR results. Sequenced samples are indicated by a vertical black line, HAdV PCR-positive samples by a red cross, HAdV PCR-negative samples by a black cross and ward stays by colored rectangles. **(A)** A31 Cluster 1, **(B)** A31 Cluster 2, **(C)** A31 Cluster 3, **(D)** A31 Cluster 4, **(E)** B3 Cluster 1, **(F)** C2 Cluster 1, **(G)** C1 Cluster 2, **(H)** C5 Cluster 3, and **(I)** F41 Cluster 1.

Previous work has shown that infections can be linked over many years (Houldcroft et al., 2018), e.g., A31 Cluster 4 potentially transmitted over a 5-year period (temporal relationship shown in Figure 3D). Using the new samples sequenced as part of this investigation, we confirmed the same with a putative transmission cluster occurring over a 3-year period (A31 Cluster 2, Figure 3B) and suggested a prolonged transmission also occurring amongst other HAdV species, e.g., B3 Cluster 1 over a 4-year period (Figure 3E).

Wards B and E were associated with all nine monophyletic clusters (Figures 2, 3). As well as sharing clinical teams, these wards are joined by the same corridor and share facilities (dirty utility, kitchen, parents' room and laundry); for this reason, they are considered one HSCT unit. Five clusters containing ward B and E patients had temporal links with each other: A31 Cluster 1, A31 Cluster 3, C1 Cluster 2, C5 Cluster 3, and F41 Cluster 1 (Figures 3A,C,G–I and Table 1), supporting nosocomial transmission.

Two clusters contained one patient with no temporal links: Pt11 in A31 Cluster 2 and Pt8 within A31 Cluster 4 (Figures 3B,D). The two remaining clusters (C2 Cluster 1, Figure 3F; B3 Cluster 1, Figure 3E) did not share any temporal links with each other.

### Confidence in Genomic Links Using Pairwise Distances

**Figure 4:**
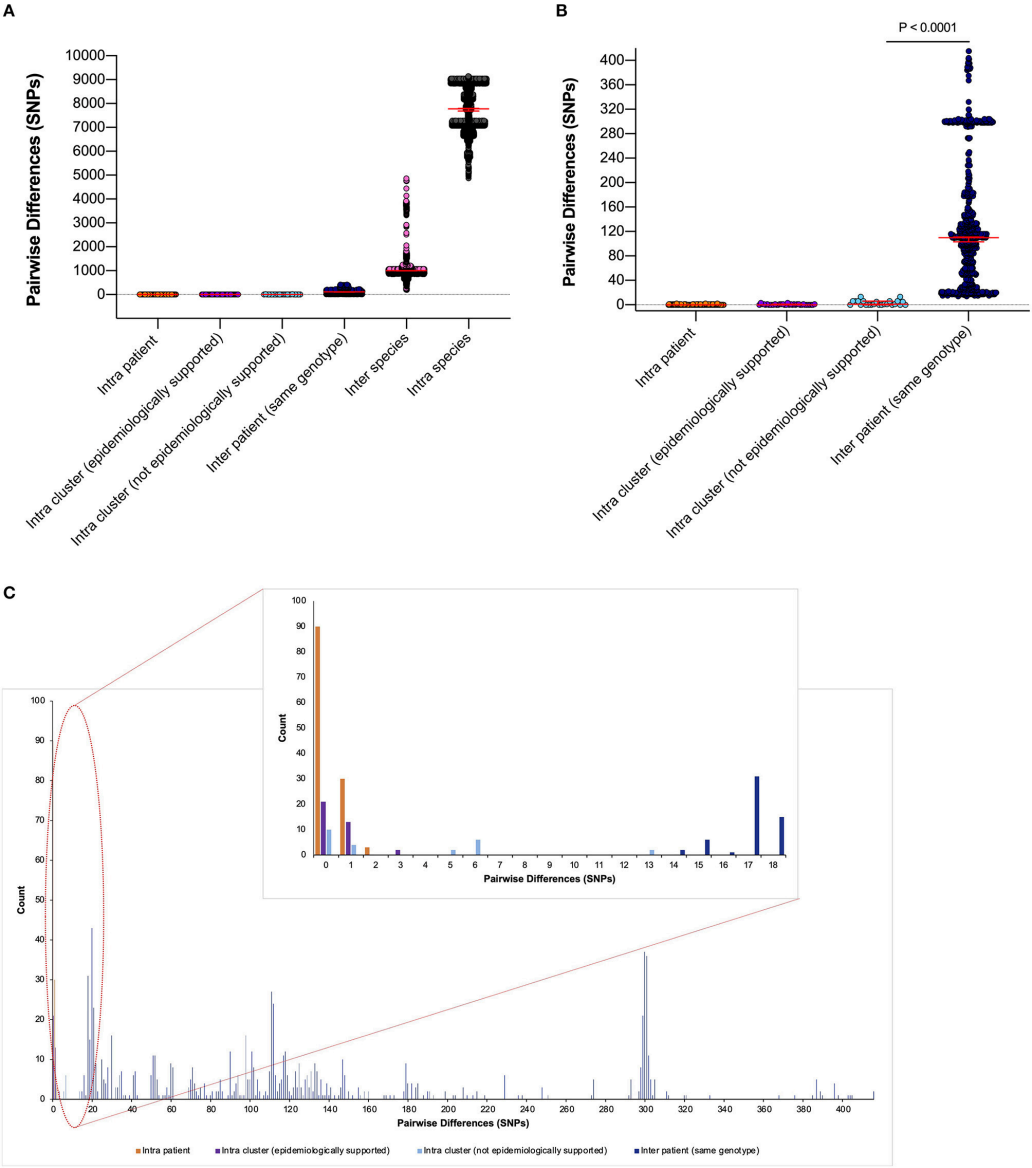
Pairwise differences equating to single-nucleotide polymorphisms (SNPs) between clinical samples included in this study. **(A)** Pairwise differences were plotted by the following categories: “intra patient,” differences between samples taken from the same patient; “intra cluster (epidemiologically supported),” differences between samples from different patients within a monophyletic cluster that are temporally linked (HAdV PCR positive whilst admitted to same/linked ward) by the admission data; “intra cluster (not epidemiologically supported),” differences between samples from different patients within a monophyletic cluster that are not linked temporally by the admission data; “inter patient (same genotype),” differences between samples from different patients within the same genotype; “inter species,” differences between samples from different patients within the same species but different genotypes and “intra species,” differences between samples from different patients between species. Median values with 95% CIs are superimposed and plotted in red. **(B)** Differences between the first four categories to aid visualization. **(C)** Highlighted precise count of pairwise differences found within the first four categories.

To quantify phylogenetic relationships, pairwise differences (single-nucleotide polymorphisms [SNPs] between aligned consensus sequences) were calculated and grouped according to their epidemiological support (Figure 4).

Epidemiologically linked monophyletic clusters were found to have ≤ 3 SNPs difference. This corroborated the number of differences previously defined for within host (≤ 2 SNPs, Figure 4C) and directly transmitted viruses (Houldcroft et al., 2018), and further supports the nosocomial transmission between clusters A31 Cluster 1, A31 Cluster 3, C1 Cluster 2, C5 Cluster 3, and F41 Cluster 1 (Table 1). Of the two patients (Pt54 and Pt60) involved in C5 Cluster 3, Pt60 died; however, this patient was admitted and HAdV PCR positive several months before Pt54 (Figure 3H), suggesting that Pt60 may have been the source of this nosocomial infection.

One monophyletic cluster, A31 Cluster 4, despite containing one patient who was not linked temporally to the other patients (Figure 3D), differed by 0–1 SNPs suggesting nosocomial transmission (Table 1). With potentially unsampled patients or environmental intermediates, it is not possible to determine the route of transmission from these patients to the other patients within these clusters.

The remaining monophyletic clusters A31 Cluster 2, B3 Cluster 1, and C2 Cluster 1 included at least one sequence separated by 6–13 SNPs. This range does not overlap with the number of SNPs found between unrelated patients of the same genotype (14–415 SNPs). However, the interpretation of the epidemiological links between cases based on the genomic data alone can be difficult because we do not currently understand the species-specific substitution rate of HAdV in chronically infected immunosuppressed patients.

B3 Cluster 1 is the monophyletic cluster with the least support, without a temporal relationship between patients (admitted and tested 4 years apart) and 13 SNPs between the two sequences (14 SNPs were found between unrelated patient samples of the same genotype within species C viruses). Only three B3 infections were identified during the study period (92 and 93 SNPs separated this cluster from unrelated Pt14). The close clustering of these samples is therefore likely to be a result of the few publicly available UK HAdV B3 sequences. Further sequencing of HAdV-B3 genotypes is therefore required to substantiate the relationship found between these two patients.

## Discussion

This study demonstrates the major threat to immunosuppressed children that HAdV presents, providing a snapshot of a larger problem as only a minority of viruses were sequenced. With the level of infection present, it is not possible to recognize HAdV outbreaks contemporaneously using the conventional PCR methods. New infections identified by the diagnostic laboratory by PCR (Figure 1) are also likely to be over- or under-represented; without genomic information, it is impossible to know whether these are genuine new infections or reactivation of clinically quiescent virus (Kosulin et al., 2016; Dhingra et al., 2019). WGS identified six patient clusters and 15 patient transmission events (patients not within an ICC) which were not identified using standard IPC investigations and whilst only focusing on high-risk patients from two wards. Rapid sequencing is now possible within 72 h. This has already been shown to impact the IPC management of SARS-CoV-2 in real time (Meredith et al., 2020) and would have impacted on the additional six patient clusters identified here.

The utility of the WGS data is entirely dependent on the quality of sequences obtained. Poor genome coverage and low read depth generate tenuous links to other patients and any SNPs identified are poorly supported. Newly designed baits improved sequencing success (85% using version 2 baits vs. 42% using version 1 baits), especially for species C viruses (Supplementary Figure 8). Species C HAdVs produce the most severe clinical manifestations amongst immunosuppressed patients, particularly those undergoing HSCT (Lion et al., 2010; Qurashi et al., 2011).

The high incidence of mixed infection (10% of patients) identified in this study highlights the superiority of WGS over PCR. Not only were patients incorrectly linked using standard methods, demonstrated by ICC 1, but HAdV PCR-positive patients would also go unnoticed if they later acquired a second HAdV co-infection. These secondary HAdV co-infections are important not only because of their role in transmission events, but also they could have different tissue tropisms, clinical consequences and provide a sentinel source of recombination (Lukashev et al., 2008; Walsh et al., 2009; Hashimoto et al., 2018).

This tertiary referral center already has robust IPC precautions in place for high-risk patients; single room isolation with en-suite facilities and environmental screening post-discharge (Cloutman-Green et al., 2015). Despite these precautions, we were still able to confirm that 21 patients (28%) were involved in a nosocomial transmission cluster, and 15 patients (20%) ordinarily would have gone unnoticed or unlinked (Table 1). Unidentified acquisition/transmission events can have clinically significant consequences including prolonged hospital stay, missed treatment opportunities and even death. Sequence data allowed us to investigate five HAdV-associated deaths as part of the Trust Patient Safety Review process. This is important for all the cases of HCAI where the patient may have come to harm. In this cohort, no death was related to a virus shown to be acquired at GOSH. The two HAdV-associated deaths that were involved in transmission events here (Pt52 and Pt60) appeared to be the index case in their respective clusters (Figures 2A,C, 3B,H).

Immediate action needs to be taken to identify the source of HAdV acquisition in these patients in order to understand and halt transmission. Index cases may acquire their HAdV infection outside of hospital, but we have evidence that widely separated samples are linked; Pt8 within A31 Cluster 4, one SNP difference (Figure 3D). This patient was immunocompromised long term (X-linked lymphoproliferative disease) which is known to facilitate prolonged viral shedding (Kosulin et al., 2016). Patient 8 was, however, discharged at least 6 months prior to related patients, and sample positivity by PCR was absent for over 3 years. This suggests ongoing, undetected nosocomial transmission by unsampled intermediates which could include the environment, other patients, visiting relatives or staff members. Outbreaks amongst vulnerable patients already harboring a high rate of HAdV positivity requires comprehensive surveillance. As a result, we have begun to implement routine HAdV WGS into a standard diagnostic algorithm to improve the clinical care.

### Conclusion

The clinical utility of WGS technology for IPC purposes has begun to be realized for a number of important pathogens (Brodrick et al., 2016; Eyre et al., 2017; Brown et al., 2019; Roy et al., 2019; Meredith et al., 2020). In this study, we have demonstrated that using a sensitive technique, HCAI and mixed infection remains a significant problem despite the application of thorough IPC containment strategies. PCR alone fails to identify HAdV co-infection and transmission events, which can have catastrophic consequences amongst high-risk patients. In order to combat this deficit, HAdV WGS is being implemented into routine diagnostics within this tertiary referral center.

## Data Availability Statement

The datasets presented in this study can be found in online repositories. The names of the repository/repositories and accession number(s) can be found in the article/Supplementary Material.

## Ethics Statement

The studies involving human participants were reviewed and approved by The National Research Ethics Service Committee London—Fulham (reference: 17/LO/1530).

## Author Contributions

CM, CH, KJR, and JB planned and designed the study. SR designed the sequencing baits. TB, CM, HD, JH, and KR provided the clinical data. CW, RW, and CM sequenced the samples. CM, SR, BM, and JG-A analyzed the data. CM, CH, and JB drafted the manuscript. All authors read, edited, and approved the final manuscript.

## Conflict of Interest

JB has declared that the adenovirus bait designs are used with permission from Agilent. The remaining authors declare that the research was conducted in the absence of any commercial or financial relationships that could be construed as a potential conflict of interest.
