# Supplementary material for: Using Whole Genome Sequences to Investigate Adenovirus Outbreaks in a Hematopoietic Stem Cell Transplant Unit
Source: Front Microbiol. 2021 Jul 2;12:667790. doi: 10.3389/fmicb.2021.667790 (PMC8284422; doi:10.3389/fmicb.2021.667790)
Supplement: Supplementary file 1 [file Presentation_1.pdf]

Supplementary Material

Contents

1.1 Supplementary Methods 2

1.1.1 Management of patients with HAdV infections 2
1.1.2 Methodology used in further analysis with illustrative examples 2
1.1.2.1 Example of a Mixed C2/A31 Infection - Patient 52_S1 3
1.1.2.2 Example of A31/A61 Cross Mapping – Patient 3_S1 3
1.1.3 Consensus alignment and phylogenetic analysis 3
1.1.4 Statistical analysis of bait performance 4

1.2 Supplementary Figures and Tables 5

Supplementary Figure 1. Schematic Illustrating HAdV Sequencing Pipeline 5
Supplementary Table 1. Sequencing statistics and diagnostic laboratory metadata for each patient sample 6
Supplementary Figure 2. Genotyping output for Patient 52_S2 12
Supplementary Table 2. Patient 52_S2 Hexon, penton and fiber sequencing statistics following simultaneous mapping with high stringency 12
Supplementary Table 3. Percent sequence identity between HAdV-A31 and A61 reference genomes 12
Supplementary Figure 3. Genotyping output for Patient 3_S1, A31 and A61 subset coverage following simultaneous mapping with high stringency 12
Supplementary Table 4. Patient 3_S1 whole genome, hexon, penton and fiber sequencing statistics following simultaneous mapping to A31 and A61 reference sequences with high stringency 13
Supplementary Figure 4. Patient 3_S1 single mapping to A31 at high stringency 13
Supplementary Figure 5. Patient 3_S1 single mapping to A61 at high stringency 13
Supplementary Table 5. Patient 3_S1 whole genome, hexon, penton and fiber sequencing statistics following single mapping with high stringency 13
Supplementary Table 6. Percent identity between Patient 3_S1 consensus sequences following single mapping to reference sequences at high stringency 13
Supplementary Figure 6. Maximum likelihood phylogeny of adenovirus full genome sequences included in this study 14
Supplementary Figure 7. Numbers of samples sequenced according to HAdV genotype using version one (A) and version two (B) baits 15
Supplementary Figure 8. HAdV full-genome sequencing outcome metrics using version one (A. to C.) and version two (D. to F.) baits 16
Supplementary Figure 9. Polymerase chain reaction cycle threshold (C_T) values for all samples sequenced with SureSelect target enrichment 18
Supplementary Figure 10. Average read depth for all non-C species (A31, B3, E4 and F41) samples using version one baits and all samples using version two baits 19
Supplementary Table 7. Table of Sequences / Accession Numbers 20

1.1 Supplementary Methods

1.1.1 Management of patients with HAdV infections

All patients who are symptomatic or HAdV positive on stool and/or respiratory PCR are isolated in single bedded rooms, with transmission-based precautions (plastic apron, disposable gloves (if risk of exposure to blood or bodily fluids) and fluid-repellent surgical face masks (FFP3 respirator, apron and eye protection for aerosol generating procedures)) in place. Appropriate samples, dependent on symptoms, are collected weekly until they are PCR negative. Any patients admitted for HSCT or with a congenital immunodeficiency are placed in protective isolation regardless of their infectious status. Each isolation room has en-suite bathroom facilities with either a positive pressure ventilated lobby leading to a neutral pressure room, or a positive pressure room without a lobby. The number of staff and visitors entering the room is kept to a minimum. The IPC team (including specialist nurses, clinical microbiologists and clinical scientists) are informed by the diagnostic laboratory of all new positive cases of HAdV.

A nosocomial outbreak is suspected on a HSCT unit when any new detection of HAdV infection is made in a child who was negative on admission screening. This excludes cases that presented with their illness within 48 hours of admission (likely community acquired infection). An outbreak control meeting is convened, and immediate infection control measures are put in place; transmission-based precautions, communal areas are closed and cleaned, nursing staff are dedicated to affected and non-affected areas, visiting is restricted, and staff are asked to risk assess family members to find out if any are symptomatic and should be excluded from visiting.

Following discharge of an HAdV positive patient terminal cleaning with 1,000ppm chlorine takes place before molecular environmental screening (cotton tipped swabs are used to take samples from the following sites: floor under the sink, clinical waste bin, chair arms, door handles (bathroom and entrance), telephone, bathroom taps, bed frame, mattress, trolley, windowsill and floor outside room). If any areas are found to be HAdV PCR positive a complete reclean and rescreen is undertaken (22).

1.1.2 Methodology used in Further Analysis with Illustrative Examples

At the genotyping stage, average read coverage and length of consensus was considered against each reference sequence. Any samples that showed a high proportion of reference genome covered (≥90%) for more than one HAdV type or, displaying a similar (≤10% difference) reference genome coverage between top hitting types were investigated further for mixed infection (Supplementary Figure 1). Sample sequences were mapped simultaneously to top hitting references thought to make up the mixed infection. This time where 90% of each read mapped with a minimum of 90% identity, samples were also mapped to individual references with 80% of each read mapped with 80% identity. Any unmapped reads were taken forward for mapping against reference sequences thought to be contained in the mixed infection. For each mapping sequencing statistics were calculated across the whole genome, in addition to the major capsid genes – the hexon, penton base and fiber – the main antigenic determinants used in typing and characterisation of new recombinant strains (37–40).

Any samples that underwent mixed analysis regardless of a single or mixed infection conclusion, had their consensus sequence extracted from a pipeline that used higher stringency parameters (90% of each read mapped with a minimum of 90% identity), to reduce cross-mapping errors influencing downstream analysis.

1.1.2.1 Example of a Mixed C2/A31 Infection - Patient 52_S1

Sequencing depth is significantly better for C2 however, the complete genome is covered for both C2 and A31, suggesting a mixed infection made up of predominantly C2 virus (Supplementary Table 2, 3 and Supplementary Figure 2).

It is highly unlikely that the minority HAdV-A31 found here is a result of laboratory contamination; the only other A31 patient sequence that was found to be identical was identified in Patient 73 (A31 Cluster 2), a mixed A31/B3 infection. Patient 52_S2 was collected 09/04/2018 and sequenced 21/05/2018, Patient 73_S1 collected 02/04/2019 and sequenced 16/09/2019.

1.1.2.2 Example of A31/A61 Cross Mapping – Patient 3_S1

A61 is a recombinant of A31 and A12: it has a unique hexon, an A31 penton and an A31 fiber. Although A61 is a recombinant of A12, A12 does not appear to generate the same cross mapping issues as A31 (Supplementary Figure 3). Reference genomes that are >90% identical become very difficult to separate out. In the case of these two viruses (sharing 97.62% sequence identity), the main differences appear to be in the hexon (Supplementary Table 3). Mapping simultaneously suggests there are regions belonging to two different viruses (Supplementary Figure 3 and Supplementary Table 4).

Mapping to single reference genomes at high stringency eliminates regions of the genome that previously yielded very low coverage and depth (Supplementary Figures 4 and 5), this suggests cross mapping rather than a mixed infection. Using the major capsid regions, the hexon gene contains the most differences (87.6% identity, Supplementary Table 3) and therefore may help with identifying mixed versus single infection. When mapped to individual references in isolation, the hexon of Pt3_S1 is closer to A31 (Supplementary Table 5). It could be that the fiber of Pt3_S1 is closer to A61, but further recombination analysis would be required to resolve this. When mapped to both reference sequences in isolation, the penton and fiber consensus sequences for this patient are identical (Supplementary Table 6). The overall genotype was therefore classified as HAdV-A31.

1.1.3 Consensus Alignment and Phylogenetic Analysis

Consensus sequences were aligned using the slow (very accurate) alignment algorithm within CLC Genomic Workbench (version 12.0.1). After applying the CLC Model Testing Tool, maximum likelihood phylogenies were constructed using the Kimura 1980 (K80), substitution model including rate variation (+G), tree topology estimation (T) and 500 bootstrap replicates. MEGA was used to calculate differences between aligned consensus sequences, pairwise differences. Here the number of alignment positions where one nucleotide within a sequence was different from another was used to define single nucleotide polymorphisms (SNPs). The Pairwise Differences tool within CLC was used to calculate the percentage of identical nucleotides between sequences in an alignment relative to each other.

Species specific phylogenies were constructed in addition to all samples and references, allowing metadata to be added and sequence clusters to be visualised in more detail.

1.1.4 Statistical Analysis of Bait Performance

The differences between estimated percentage reference genome coverage, average read depth and percent OTRs and in PCR C_T values between C and non-C species were tested using the Mann-Whitney un-paired nonparametric test. The relationships between PCR cycle threshold (C_T) values and percentage reference genome coverage, average read depth and percent OTRs were assessed using Spearman’s correlations.

1.2 Supplementary Figures and Tables

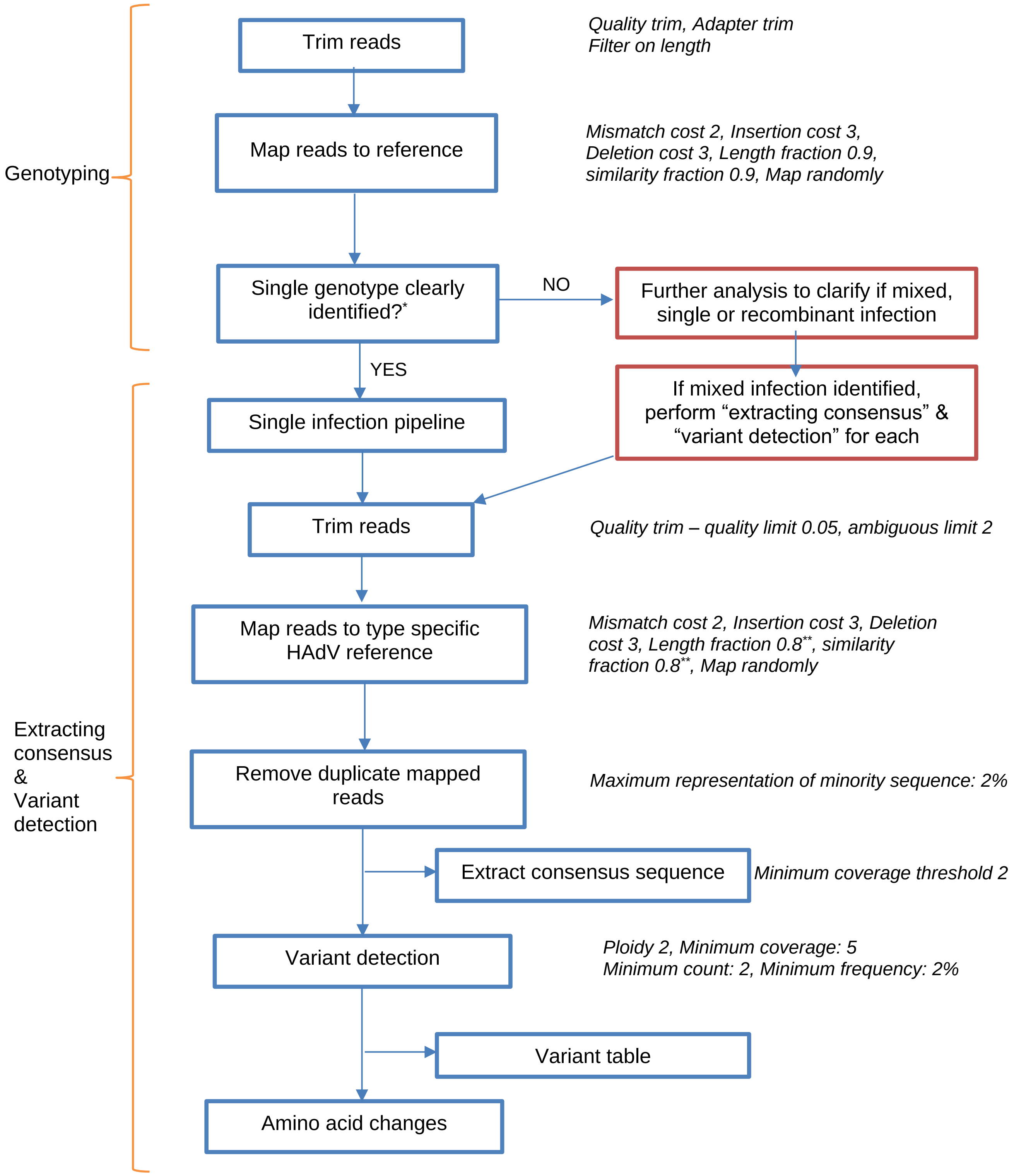

Supplementary Figure 1. Schematic Illustrating HAdV Sequencing Pipeline.
Italicised text summarises workbench settings
*Samples with a high proportion of reference genome covered (≥90%) for more than one HAdV genotype, or <10% difference between top hitting types
**Samples pre-analysed for mixed infection processed with a length fraction and similarity fraction of 0.9

Supplementary Table 1. Sequencing statistics and diagnostic laboratory metadata for each patient sample.

Patient	Underlying diagnosis	Sample Code	Sample type	Bait version	Possible species	VL (copies/ml)	C_T	Coverage	Depth	%OTRs	Final Species
1	Scoliosis, developmental delay, epilepsy	Pt1_S1	Blood	1	E4	4,010,170	26	99%	317.32	11.58	E4
		Pt1_S2	Blood	1	E4	20,000,001	22	99%	1163.26	41.85	E4
2	Primary immunodeficiency	Pt2_S1	Blood	1	C1	932,130	28	78%	66.82	2.63	C1
3	Primary immunodeficiency	Pt3_S1	Blood	1	A31	167,847	30	100%	751.55	38	A31
4	Primary immunodeficiency	Pt4_S1	Faeces	1	Failed (C1)		31	13%	0.35	0.01	Failed
5	ALL	Pt5_S1	Blood	1	C89	699,635	28	82%	124.98	2.86	C89
		Pt5_S2	Blood	1	C89	773,056	28	79%	52.72	1.24	C89
		Pt5_S3	Blood	1	C89	1,134,740	27	75%	40.08	1.06	C89
6	Evans syndrome	Pt6_S1	Blood	1	A31	152,741	30	100%	787.85	33.81	A31
7	Severe combined immunodeficiency	Pt7_S1	Eye swab	1	C1		30	84%	127.03	4.01	C1
8	Primary immunodeficiency	Pt8_S1	Blood	1	A31	78,190	32	100%	2404.71	78.92	A31
		Pt8_S2	Blood	1	A31	6,116	35	100%	2049.04	63.87	A31
9	ALL	Pt9_S1	Blood	1	C5	55,406	32	56%	4.91	0.15	C5
		Pt9_S2	Blood	1	C5	148,412	31	72%	21.73	0.72	C5
		Pt9_S3	Blood	1	C5	159,334	30	79%	95.8	2.95	C5
		Pt9_S4	Blood	1	C5	175,512	30	81%	89.83	2.73	C5
		Pt9_S5	Blood	1	C5	51,527	32	61%	11.5	0.34	C5
		Pt9_S6	Blood	1	C5	40,729	32	50%	4	0.24	C5
		Pt9_S7	Blood	1	C5	103,556	32	69%	23.33	1.12	C5
		Pt9_S8	Blood	1	C5	582,874	29	72%	31.67	1.05	C5
		Pt9_S9	Blood	1	C5	336,548	29	72%	26.06	0.88	C5
		Pt9_S10	Blood	1	C5	441,445	29	69%	24.56	1.15	C5
		Pt9_S11	Blood	1	C5	509,127	28	74%	34.27	1.42	C5
		Pt9_S12	Blood	1	C5	428,489	29	77%	77.65	2.8	C5
		Pt9_S13	Blood	1	C5	1,727,720	27	82%	147.48	5.96	C5
		Pt9_S14	Blood	1	C5	489,344	29	82%	127.53	4.84	C5
		Pt9_S15	Blood	1	C5	303,814	29	67%	23.98	0.98	C5
		Pt9_S16	Blood	1	C5	201,184	30	66%	23.02	0.87	C5
10	Primary immunodeficiency	Pt10_S1	Blood	1	C89	1,264,060	27	79%	127.5	7.25	C89
		Pt10_S2	Blood	1	C89	133,306	31	70%	32.5	1.44	C89
11	T-cell immunodeficiency	Pt11_S1	Blood	1	A31	30,591,600	22	100%	4319.26	77.79	A31
		Pt11_S2	Blood	1	A31		21	100%	5116.98	78.3	A31
12	Severe Combined Immunodeficiency	Pt12_S1	Blood	1	C1	4,292	36	49%	4.99	0.32	C1
13	Down syndrome/trisomy 21, Hirschsprung's disease	Pt13_S1	Faeces	1	F41		21	100%	717.09	65.58	F41

14	X-linked Severe Combined Immunodeficiency	Pt14_S1	NPA	1	B3/B66		26	100%	1732.74	44.68	B3
15	ALL	Pt15_S1	Urine	1	Failed	1,279	37	-	-	-	Failed
		Pt15_S2	Blood	1	C5	93,126	31	48%	3.65	0.15	C5
		Pt15_S3	Blood	1	C5	95,940	32	50%	4.37	0.15	C5
		Pt15_S4	Blood	1	C5	215,793	30	69%	27.04	0.93	C5
		Pt15_S5	Blood	1	C5	92,183	32	69%	23.67	0.93	C5
16	Undiagnosed immunodeficiency	Pt16_S1	Urine	1	C5 / C1	20,000,001	21	97% (C5)<br>97% (C1)	2923.71 /<br>2230.04	67.9<br>/ 52.71	C5 / C1
		Pt16_S2	Blood	1	C1 / C5	10,682,000	24	92% (C1)<br>92% (C5)	903.43 /<br>820.04	30.37 /<br>27.71	C1 / C5
		Pt16_S3	Blood	1	C5 / C1	20,000,001	21	97% (C5)<br>97% (C1)	2489.37 /<br>2245.69	69.32 /<br>61.62	C5 / C1
		Pt16_S4	Urine	1	C1 / C5	16,597,100	23	98% (C1)<br>98% (C5)	3103.90 /<br>2693.95	59.29 /<br>51.59	C1 / C5
		Pt16_S5	Blood	1	C5 / C1	178,674,000	20	98% (C5)<br>98% (C1)	2980.60 /<br>2685.45	80.58 /<br>73.34	C5 / C1
		Pt16_S6	Urine	1	C5 / C1	68,486	32	28% (C5)<br>23% (C1)	0.79 / 0.64	0.04 /<br>0.03	C5 / C1
		Pt16_S7	Blood	1	C5 / C1	20,000,001	18	98% (C5)<br>98% (C1)	2224.03 /<br>1989.97	80.56 /<br>72.14	C5 / C1
		Pt16_S8	Blood	1	C5 / C1	20,000,001	17	100% (C5)<br>100% (C1)	4612.51 /<br>4042.38	84.65 /<br>73.77	C5 / C1
		Pt16_S9	Blood	1	C5 / C1	20,000,001	17	100% (C5)<br>100% (C1)	6258.15 /<br>5282.94	86.52 /<br>73.86	C5 / C1
17	Wilms' Tumour	Pt17_S1	Throat swab	1	C1		32	71%	14.97%	0.71	C1
18	Primary Immunodeficiency	Pt18_S1	Urine	1	A31	16,673	34	100%	67.91	2.37	A31
		Pt18_S2	Blood	1	A31	55,525	32	100%	250.95	9.15	A31
19	ALL	Pt19_S1	Blood	1	C2	70,523	32	72%	30.7	0.96	C2
		Pt19_S2	Blood	1	C2	158,917	31	72%	36.38	1.28	C2
		Pt19_S3	Blood	1	C2	59,267	32	49%	4.84	0.17	C2
		Pt19_S4	Blood	1	C2	56,325	32	37%	2.4	0.07	C2
		Pt19_S5	Blood	1	C2	488,595	28	64%	12.28	0.51	C2
		Pt19_S6	Blood	1	C2	765,653	27	72%	29.79	1.18	C2
		Pt19_S7	Urine	1	C2		27	92%	757.52	17.26	C2
		Pt19_S8	Blood	1	C2	6,000,230	25	89%	880.36	25.18	C2
		Pt19_S9	Blood	1	C2	34,149,600	22	88%	637.27	16.54	C2
		Pt19_S10	Blood	1	C2	34,947,600	22	92%	1793.86	47.31	C2
		Pt19_S11	Blood	1	C2	42,970,500	22	93%	1713.64	55	C2
		P19_S12	Eye swab	1	C2	728,939	28	90%	290.31	7.20	C2
		Pt19_S13	Blood	1	C2	85,855,600	21	94%	2506.27	66.43	C2

		Pt19_S14	Blood	1	C2	26,560,200	23	99%	2058.96	52.32	C2
		Pt19_S15	Blood	1	C2	75,178,500	21	100%	3137.82	72.64	C2
		Pt19_S16	Blood	1	C2	5,274,900	25	100%	3105.74	76.13	C2
		Pt19_S17	Blood	1	C2	63,197,600	21	100%	2488.76	71.11	C2
		Pt19_S18	Blood	1	C2	20,407,000	23	98%	1456.96	38.8	C2
		Pt19_S19	Ascitic fl.	1	C2	12,730,700	24	96%	1853.07	54.47	C2
		Pt19_S20	Blood	1	C2	1,650,640	26	94%	331.93	9.13	C2
		Pt19_S21	Swab	1	C2		24	96%	2321.12	56.27	C2
		Pt19_S22	Blood	1	C2	577808	28	85%	93.79	4.12	C2
		Pt19_S23	Blood	1	C2	67425	31	70%	21.3	0.55	C2
		Pt19_S24	ET asp.	1	C2		25	93%	1151.69	24.17	C2
		Pt19_S25	Other	1	C2	287,760	29	72%	26.5	0.68	C2
		Pt19_S26	Urine	1	C2	40,844	33	46%	3.59	0.16	C2
		Pt19_S27	Blood	1	C2	13,699	35	44%	3.18	0.21	C2
		Pt19_S28	Blood	1	Failed	36,933	32	-	-	-	Failed
		Pt19_S29	Blood	1	C2	71,985	31	48%	4.58	0.16	C2
20	AML	Pt20_S1	Urine	1	C5	97,322	31	53%	4.08	0.22	C5
21	Primary Immunodeficiency	Pt21_S1	Blood	1	C5	6,401	35	23%	0.63	0.02	C5
22	Haemophagocytic lymphohistiocytosis	Pt22_S1	Blood	1	C1 / C5	181,734	30	73% (C1) 73% (C5)	36.27 / 34.91	1.27 / 1.23	C1 / C5
		Pt22_S2	Blood	1	C1 / C5	93,957	31	67% (C1) 68% (C5)	17.43 / 16.74	0.56 / 0.54	C1 / C5
23	Cystic fibrosis	Pt23_S1	NPA	1	C1		32	52%	5.88	0.21	C1
		Pt23_S2	BAL	1	C1		24	89%	480.46	15.21	C1
24	Primary immunodeficiency	Pt24_S1	Blood	1	A31	268,045	29	100%	1198.85	35.69	A31
		Pt24_S2	Blood	1	A31	120,000	30	100%	701.22	26.72	A31
		Pt24_S3	Blood	1	A31	582,127	28	100%	1844.92	50	A31
		Pt24_S4	Blood	1	A31	2,056,900	27	100%	1877.33	40.07	A31
25	Spinal neuroblastoma	Pt25_S1	Urine	1	C1	20,000,001	19	100%	3685.3	73.33	C1
26	Primary immunodeficiency	Pt26_S1	NPA	1	C1		21	99%	3502.61	79.18	C1
27	No information	Pt27_S1	Blood	1	B3 / B66	20,000,001	17	100%	3744.13	85.7	B3
		Pt27_S2	Blood	1	B3 / B66	1,233,050	27	100%	2826.07	80.71	B3
28	Primary immunodeficiency	Pt28_S1	Blood	1	C5 / C2	206,861	30	82%	456.39	2.01	C5
29	AML	Pt29_S1	Throat swab	1	A31	20,200	34	100%	182.67	4.2	A31
30	Metastatic neuroblastoma	Pt30_S1	Urine	1	F41	613,713	28	100%	513.7	14.36	F41
31	Burkitt’s lymphoma	Pt31_S1		1	Failed			-	0.38	0.01	Failed
32	Down syndrome/trisomy 21	Pt32_S1	NPA	1	C2		30	50%	4.43	0.12	C2
33	ALL	Pt33_S1	Urine	1	C5	21,640	33	68%	9.12	0.28	C5

		Pt33_S2	Faeces	1	C5		29	55%	4.53	0.28	C5
34	Primary immunodeficiency	Pt34_S1	BAL	1	C89		18	99%	10472.65	51.06	C89
		Pt34_S2	Blood	1	C89	2,552		48%	2.97	0.02	C89
		Pt34_S3	Blood	1	C89	7,739		61%	10.95	0.11	C89
		Pt34_S4	Blood	1	C89	11,047		46%	3.71	0.03	C89
		Pt34_S5	Faeces	1	C89		31	65%	20.58	0.1	C89
		Pt34_S6	Faeces	1	C89		31	67%	10.48	0.09	C89
		Pt34_S7	Faeces	1	C89		31	71%	11.02	0.08	C89
35	Neurofibromatosis	Pt35_S1	Urine	1	C2	5,710	35	58%	5.79	0.15	C2
		Pt35_S2	Blood	1	C2	308,628	30	81%	107.08	4.21	C2
		Pt35_S3	Blood	1	C2	85,561	31	79%	62.99	2.4	C2
36	Infection	Pt36_S1	Faeces	1	C1		33	90%	192.37	5.5	C1
37	HLH	Pt37_S1	NPA	1	Failed		37		0.19	0.02	Failed
38	Primary immunodeficiency	Pt38_S1	Blood	1	C1	317,524	30	59%	7.02	0.06	C1
39	Primary immunodeficiency	Pt39_S1	Blood	2	C1	15,392	34	100%	602.5	26.14	C1
		Pt39_S2	Blood	2	C1	279,839	30	100%	3461.15	70.06	C1
		Pt39_S3	Blood	2	C1	848,239	30	100%	5479.91	93.98	C1
		Pt39_S4	Blood	1	C1	974,367	28	86%	159.31	4.03	C1
		Pt39_S5	Blood	1	C1	1,015,820	28	89%	466.42	4.59	C1
40	Severe combined immunodeficiency	Pt40_S1	Blood	2	C1	10,613	35	100%	2559.34	77.04	C1
		Pt40_S2	Blood		As above	6,857	35	Samples	combined	-	C1
		Pt40_S3	Blood	1	C1	134,741	32	83%	59.09	0.58	C1
		Pt40_S4	Blood	1	C1	69,376	32	86%	100.29	0.94	C1
41	Severe combined immunodeficiency	Pt41_S1	Blood	1	A31 / A61	48,484	32	100%	568.94	21.7	A31
		Pt41_S2	Blood	1	A31 / A61	Not available		100%	1565.18	41.63	A31
		Pt41_S3	Faeces	2	A31 / A61		22	100%	7951.03	76.41	A31
		Pt41_S4	Blood	2	A31 / A61	630,711	28	100%	5948.76	70.65	A31
		Pt41_S5	Blood	2	Failed	164,479	31	-	-	-	Failed
		Pt41_S6	Blood	2	A31 / A61	731,195	28	100%	6734.5	75.39	A31
42	Suspected immunodeficiency	Pt42_S1	Blood	1	C1	20,000,001	19	97%	4233.23	91.35	C1
		Pt42_S2	Blood	2	C1	94,794,200	21	100%	11622.89	93.74	C1
		Pt42_S3	Blood	2	C1	167,124	30	100%	1480.81	46.43	C1
		Pt42_S4	Blood	2	Failed	-			7.49	0.22	Failed
		Pt42_S5	Blood	2	C1	23666	33	72%	7.83	0.51	C1
43	Unknown	Pt43_S1	Faeces	2	C89		33	98%	58.92	0.97	C89

44	Unknown	Pt44_S1	Faeces	2	C1		25	100%	4850.23	80.82	C1
		Pt44_S2	NPA	2	C1		31	94%	49.26	0.76	C1
45	Unknown	Pt45_S1	Faeces	2	C2		33	100%	155.63	2.26	C2
46	Unknown	Pt46_S1	Faeces	2	A31 / A61		18	100%	18289.6	95.94	A31
47	Unknown	Pt47_S1	Faeces	2	C1		33	100%	891.27	26.19	C1
48	Unknown	Pt48_S1	Faeces	2	C5 / C1		29	100% (C5) 97% (C1)	2977.12 / 2628.81	44.92	C5
49	Unknown	Pt49_S1	Faeces	2	C1		33	100%	530.34	13.74	C1
50	Unknown	Pt50_S1	Faeces	2	Failed		27	-	-	-	Failed
51	Unknown	Pt51_S1	Faeces	2	C89		33	98%	51.05	1.61	C89
52	Primary immunodeficiency	Pt52_S1	Blood	2	C2 / A31	31,607	32	100% (C2) 100% (A31)	1951.49 / 186.43	72.47 / 4.22	C2 / A31
		Pt52_S2	Faeces	2	C2 / A31		15	100% (C2) 100% (A31)	16848.87 / 1626.54	90.94 / 8.12	C2 / A31
53	ALL	Pt53_S1	Blood	2	C5	145305	30	100%	1630.98	36.56	C5
		Pt53_S2	Blood	2	C5	20857200	23	100%	7867.9	96.23	C5
		Pt53_S3	Blood	2	C5	13008100	23	100%	5489.97	82.79	C5
54	Primary immunodeficiency	Pt54_S1	Faeces	2	C5		24	100%	4763.19	97.3	C5
55	Severe combined immunodeficiency	Pt55_S1	Faeces	2	A31 / A61		17	100%	8274.81	99	A31
56	ALL	Pt56_S1	Faeces	2	C1		22	99%	3401.15	87.3	C1
57	ALL	Pt57_S1	Faeces	2	A31 / A61		22	100%	5969.73	98.92	A31
58	X-linked lymphoproliferative disease	Pt58_S1	Faeces	2	C2 / A31		24	100% (C2) 100% (A31)	2968.95 / 479.17	90.53 / 11.86	C2 / A31
59	Primary immunodeficiency	Pt59_S1	Faeces	2	B11 / B35		26	100% (B11) 98% (B35)	4743.24 / 4682.92	84.11	B11
60	Primary immunodeficiency	Pt60_S1	Faeces	2	C5		22	100%	4129.2	77.43	C5
61	ALL	Pt61_S1	Faeces	2	C2/C6		22	100% (C2) 97% (C6)	3930.05 / 3630.77	98.23	C2
62	Severe combined immunodeficiency	Pt62_S1	Faeces	2	A31/A61		15	100%	7715.12	95.53	A31
63	HLH	Pt63_S1	Faeces	2	F41		32	100%	731.64	18.84	F41
64	Juvenile myelomonocytic leukaemia	Pt64_S1	Faeces	2	C2/A31/C89		33	100% (C2) 77% (A31)	116.15 / 16.32	2.27	C2 / A31
65	Inflammatory Bowel Disease	Pt65_S1	Faeces	2	F41		24	100%	4835.63	96.26	F41
66	X-linked lymphoproliferative disease	Pt66_S1	Faeces	2	C2		25	99%	203.50	79.25	C2
67	Primary Immunodeficiency	Pt67_S1	Faeces	2	C2 / C89		26	100%	1134.37	30.06	C2
68	ALL	Pt68_S1	Faeces	2	A31 / A61		21	100%	6149.73	95.4	A31

69	Primary immunodeficiency	Pt69_S1	Faeces	2	A31 / C2 / A61	30	100% (A31) 100% (C2)	268.69 / 88.14	5.76 / 8.14	A31 / C2
70	Aplastic anaemia	Pt70_S1	Faeces	2	A31 / A61	23	100%	4857.84	97.72	A31
71	Severe combined immunodeficiency	Pt71_S1	Faeces	2	F41	20	100%	4835.39	97.78	F41
72	Primary Immunodeficiency	Pt72_S1	Faeces	2	C1	23	100%	2795.17	95.76	C1
73	Severe aplastic anaemia	Pt73_S1	Faeces	2	A31 / B3 / A61	17	100% (A31) 100% (B3)	5808.74 / 372.48	95.55 / 7.21	A31 / B3
74	HLH	Pt74_S1	Faeces	2	C1 / C2	25	99%	2766.26	91.39	C1

Abbreviations: VL, viral load; C*T*, cycle threshold; %OTRs, percent on-target reads; ALL, acute lymphoblastic leukaemia; AML, acute myeloid leukaemia; HLH, haemophaogcytic lymphohistiocytosis; NPA, nasopharyngeal aspirate; BAL, broncho-alveolar lavage; ET, endotracheal
Cells highlighted in red indicate patients that died from or in association with overwhelming HAdV infection

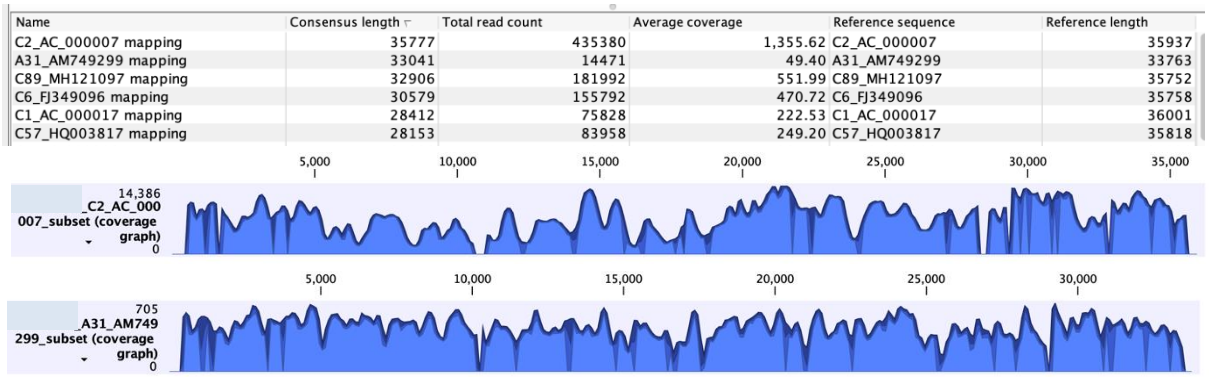

Supplementary Figure 2. Genotyping output Pt52_S2: good coverage for HAdV-C2 however, near complete consensus length generated for both C2 and A31. C2 and A31 Subset Coverage, following simultaneous mapping with high stringency.

Supplementary Table 2. Hexon, penton and fiber sequencing statistics following simultaneous mapping with high stringency.

		HAdV-A31			HAdV-C2		
	**Gene**	**Identity (%)**	**Average Coverage (%)**	**Average Depth**	**Identity (%)**	**Average Coverage (%)**	**Average Depth**
**Pt52_S2**	**Hexon**	99.9	100	456.9	99.9	100	9916.5
	**Penton**	99.6	100	439.9	99.9	100	9280.1
	**Fiber**	97.6	100	502.2	99.9	100	8510.1
	**Whole Genome**	99.68	100	444.1	99.72	100	7672.2

Supplementary Table 3. Percent sequence identity between HAdV-A31 and A61 reference genomes.

		HAdV-A61
	**Gene**	**Identity (%)**
**HAdV-A31**	Hexon	87.6
	Penton	99.1
	Fiber	98.1
	Whole Genome	97.6

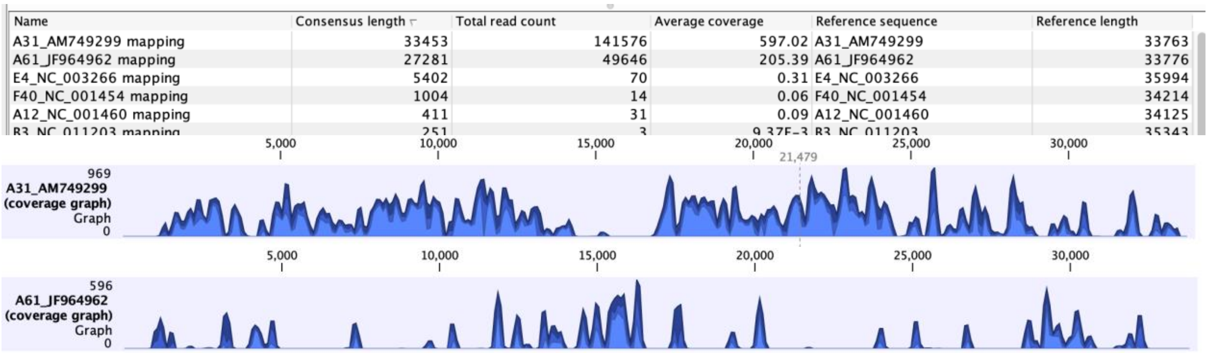

Supplementary Figure 3. Genotyping output for Pt3_S1, A31 and A61 subset coverage following simultaneous mapping with high stringency.

Supplementary Table 4. Whole genome, hexon, penton and fiber sequencing statistics following simultaneous mapping to A31 and A61 reference sequences with high stringency.

		HAdV-A31			HAdV-A61		
	**Gene**	**Identity (%)**	**Average Coverage (%)**	**Average Depth**	**Identity (%)**	**Average Coverage (%)**	**Average Depth**
**Pt3_S1**	**Hexon**	**99.2**	**100**	**319.72**	37.5	39	41.61
	**Penton**	98.7	75	63.61	98.7	76	93.91
	**Fiber**	84.0	79	94.11	**98.4**	**97**	**133.05**
	**Whole Genome**	**99.5**	**99**	**228.1**	99.59	81	44.5

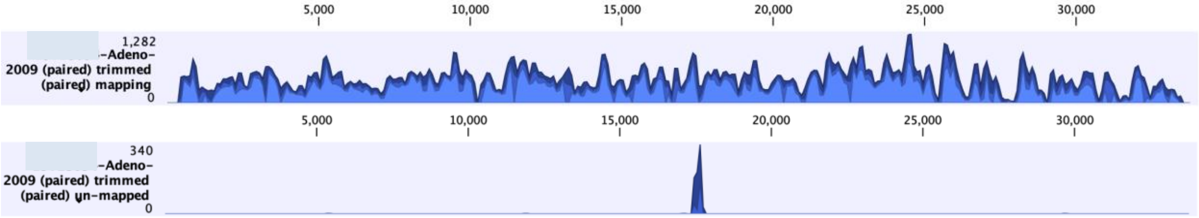

Supplementary Figure 4. Single mapping of Pt3_S1 to A31 at high stringency (top), unmapped reads mapped to A61 with high stringency (bottom). There are zero regions with coverage >25% and very poor depth.

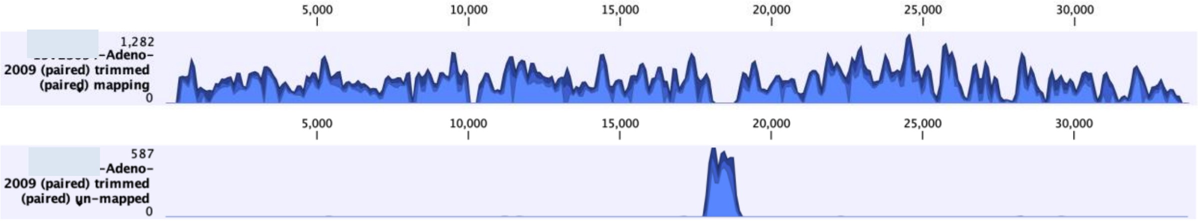

Supplementary Figure 5. Single mapping of Pt3_S1 to A61 at high stringency (top), unmapped reads mapped to A31 with high stringency (bottom). There is one region with average coverage of 44% and average depth 145.61, the hexon gene.

Supplementary Table 5. Whole genome, hexon, penton and fiber sequencing statistics following single mapping with high stringency.

		HAdV-A31			HAdV-A61		
	Gene	Identity (%)	Average Coverage (%)	Average Depth	Identity (%)	Average Coverage (%)	Average Depth
Pt3_S1	hexon	**99.67**	**100**	**396.54**	97.23	68	247.54
	penton	99.28	100	355.52	99.28	100	355.33
	fiber	98.33	100	312.17	99.16	100	312.68
	WG	**99.41**	**100**	**396.5**	98.58	97	382.4

Supplementary Table 6. Percent identity between Patient 3_S1 consensus sequences following single mapping to reference sequences at high stringency.

		Pt3_S1_A61
	**Gene**	**Identity (%)**
**Pt3_S1_A31**	Hexon	99.5
	Penton	100
	Fiber	100
	Whole Genome	99.86

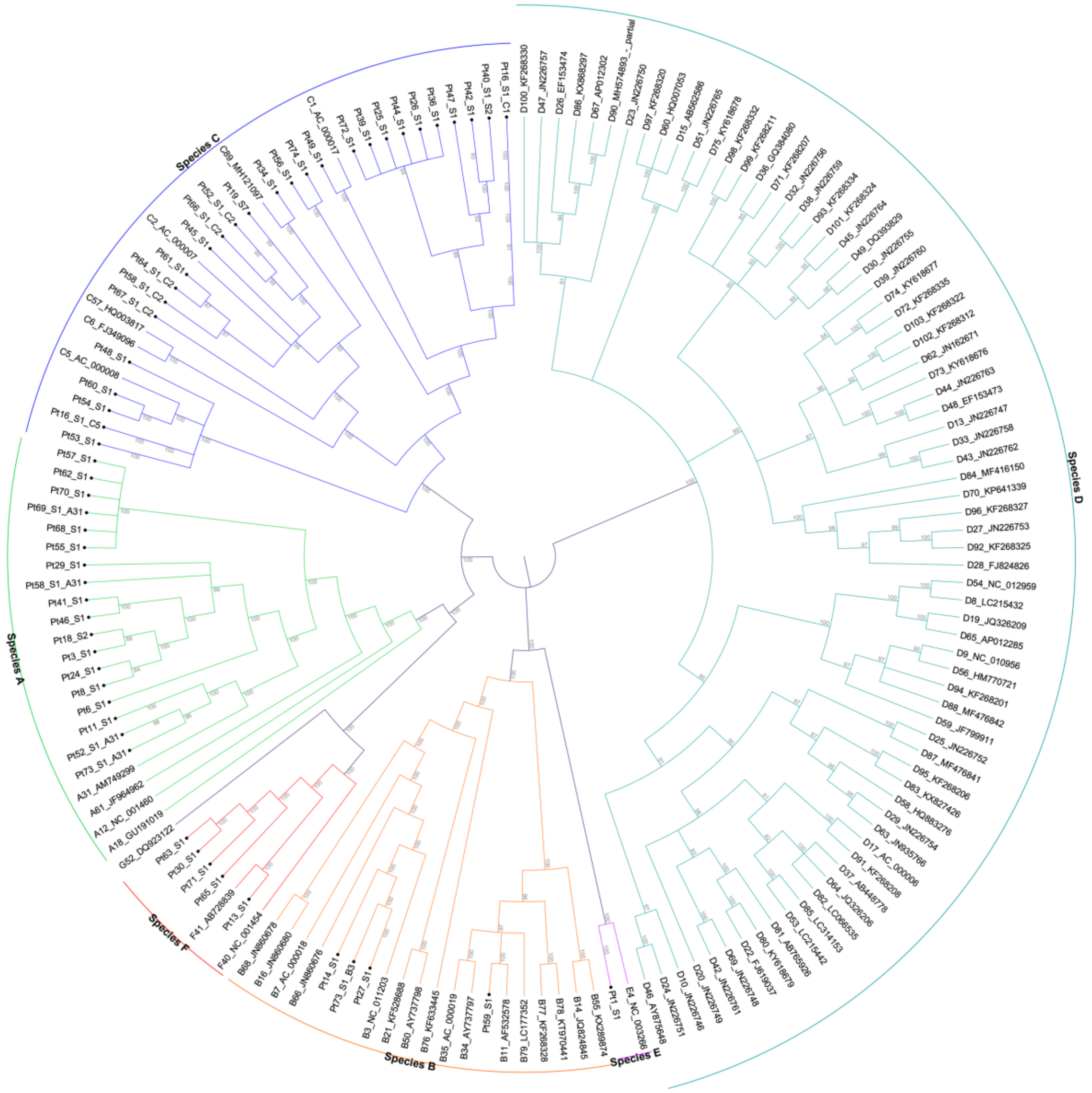

Supplementary Figure 6. Maximum likelihood phylogeny of adenovirus full genome sequences included in this study. Subtrees are highlighted in different colours according to HAdV species (A-G). Sequences were aligned using CLC Genomics Workbench (version 12.0.1) and maximum likelihood phylogenies generated using RAXML black box, 500 bootstraps. Clinical samples, marked by a dot, are labelled according to their anonymised patient number (PtX) and specimen number (_SX). Additional samples from sequentially sampled patients have been collapsed. Reference sequences are labelled according to their genotype and GenBank Accession (i.e., C5_AC_000008). Bootstrap support values of greater than 80% are shown.

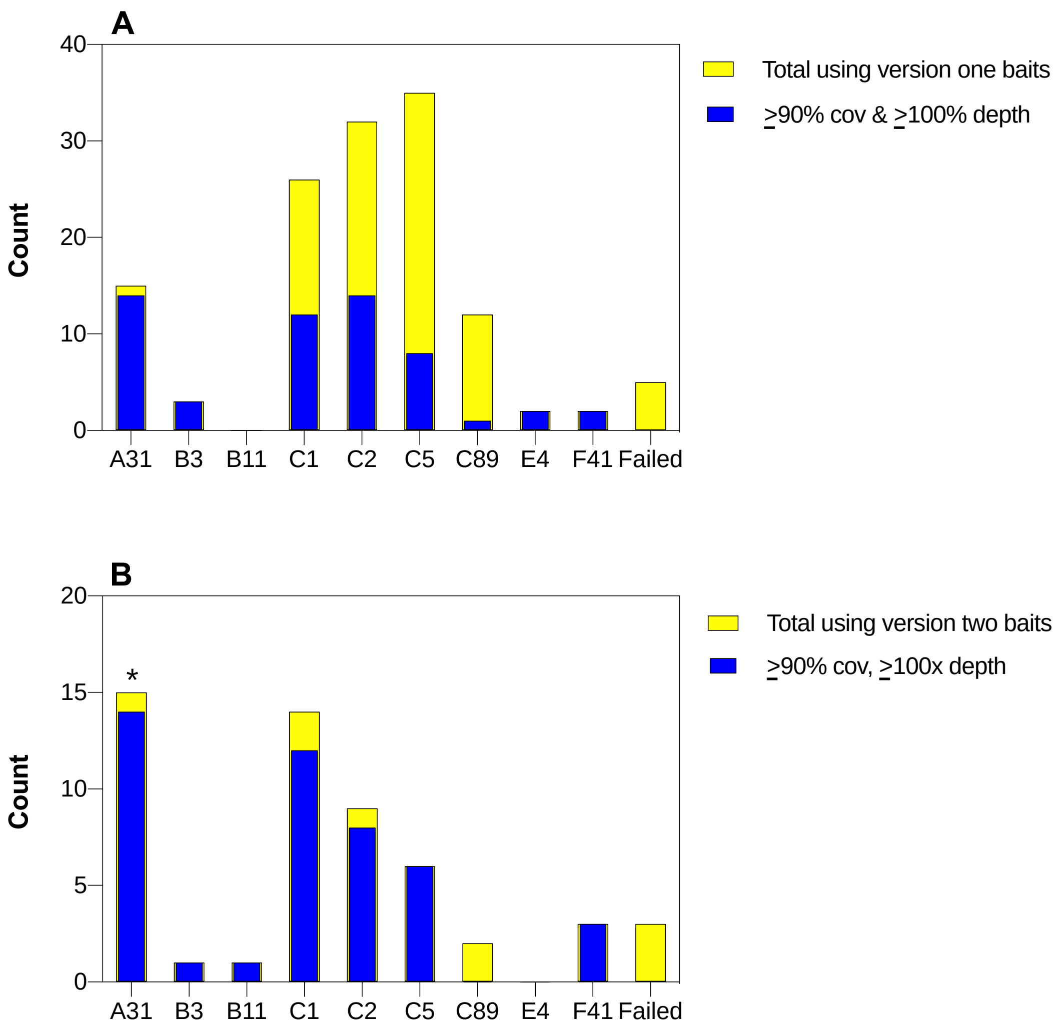

Supplementary Figure 7. Numbers of samples sequenced according to HAdV genotype using version one (**A**) and version two (**B**) baits. The proportion of samples achieving accurate near-complete genomes defined as, $\geq$90% coverage and $\geq$100-fold read depth, were improved using version two baits. *The one HAdV-A31 sequenced using version two baits that failed to achieve >90% coverage and >100x depth, made up a minority population of a mixed HAdV infection.

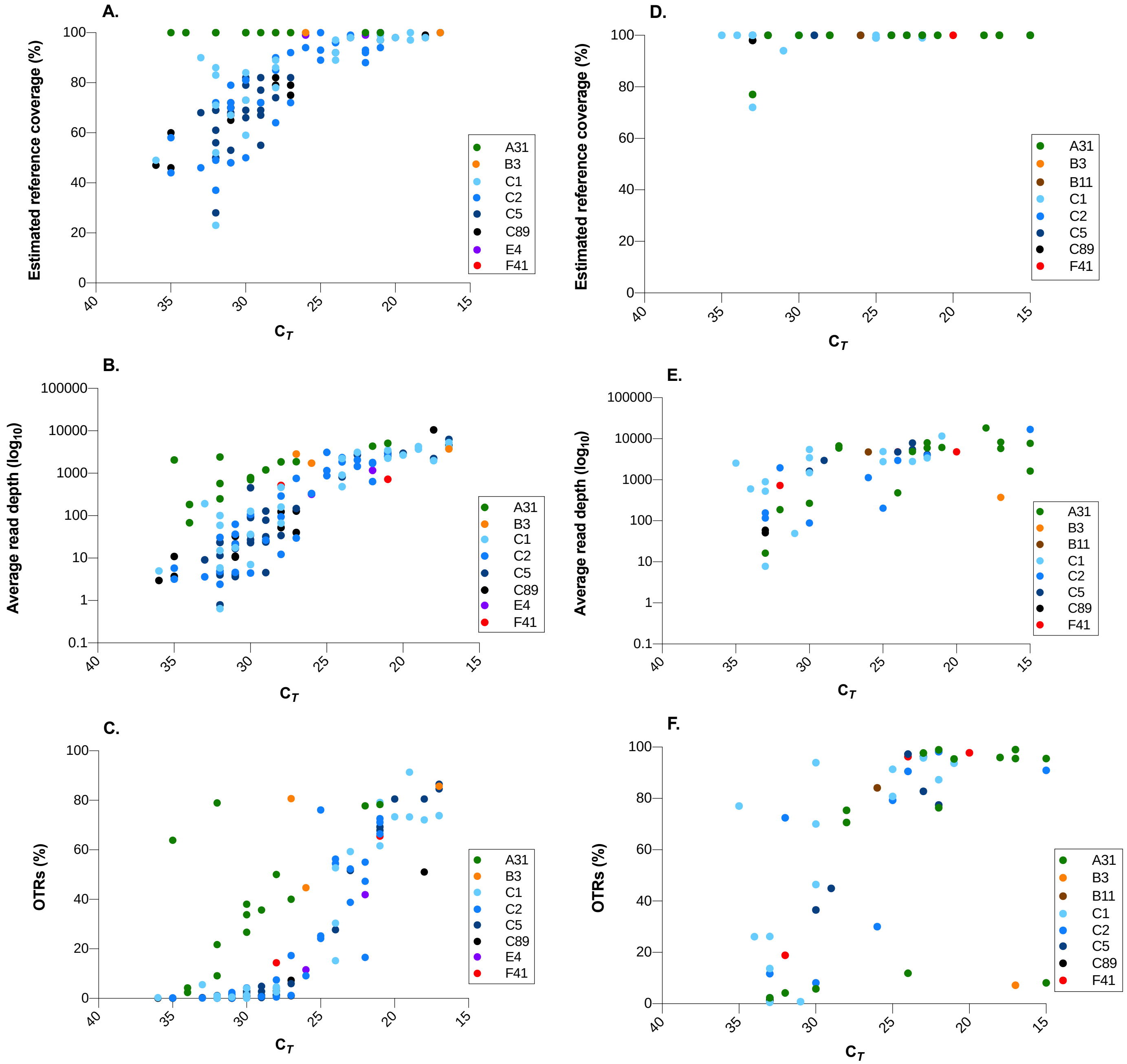

Supplementary Figure 8. HAdV full-genome sequencing outcome metrics using version one (**A** to **C**) and version two (**D** to **F**) baits. Failed samples were excluded (*n* = 7). Polymerase chain reaction cycle threshold (C_T) values plotted in reverse to illustrate a lower C_T value equating to a higher pathogen load. Statistical significance was calculated using the Mann-Whitney un-paired nonparametric test and correlation, using Spearman's correlation within GraphPad Prism, version 8.3.0 for mac OS, GraphPad Software, San Diego, California USA, www.graphpad.com. The previously published relationship between C_T value and genome coverage for species C viruses (genotypes C1, C2, C5 and C89) that was not observed for non-C species (A31, B3, E4 and F41) (A), is not seen using version two baits (B). Significantly lower estimated reference coverage (*P* = <0.0001) and OTRs (*P* = <0.0001) previously found in species C viruses when compared with non-C

species, using version one baits (**A** and **C** respectively), was not identified with version two baits (*P* = 0.1306 and *P* = 0.2700 respectively), **D** and **F**. Although improved, average read depth remained significantly lower for species C viruses compared with non-C species regardless of the baits used (*P* = 0.0002 version one baits, versus *P* = 0.05, version two baits), **B** and **E**. Significant correlations were found between C_T value and read depth, and C_T value and OTRs for version one or version two baits (read depth; version one baits *r* = 0.80, *n* = 126, *P* = <0.0001, version two baits *r* = 0.67, *n* = 55, *P* = <0.0001, OTRs; version one baits *r* = 0.79, *n* = 126, *P* = <0.0001, version two baits *r* = 0.66, *n* = 55, *P* = <0.0001).
Abbreviation: OTR, on-target reads.

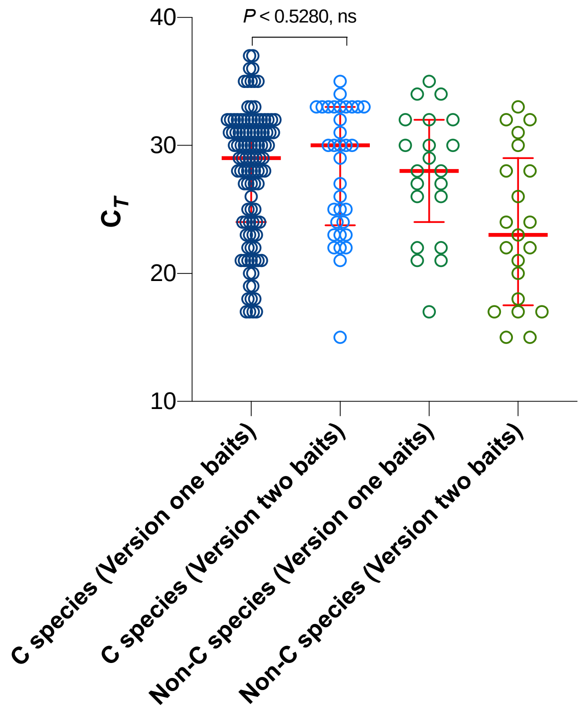

Supplementary Figure 9. Polymerase chain reaction cycle threshold (C_T) values for all samples sequenced with SureSelect target enrichment. Red horizontal lines equate to median values and vertical red bars, interquartile range. Statistical significance was calculated using the Mann-Whitney un-paired nonparametric test within GraphPad Prism, version 8.3.0 for mac OS, GraphPad Software, San Diego, California USA, www.graphpad.com. No significant difference was found between C_T value and therefore input pathogen titre amongst species C viruses (genotypes C1, C2, C5 and C89) when comparing version one versus version two baits.

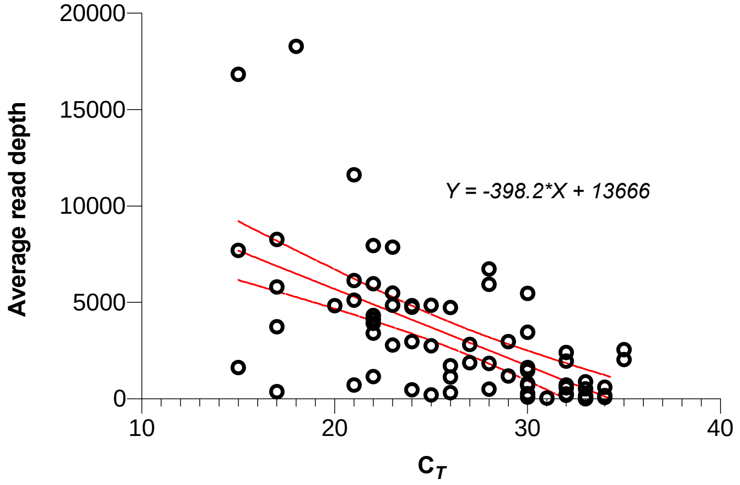

Supplementary Figure 10. Average read depth for all non-C species (A31, B3, E4 and F41) samples using version one baits and all samples using version two baits. The fitted linear regression model is shown plotted in red with 95% confidence intervals. The dependent variable *y* is the average read depth and the independent variable *x* is the PCR C_T value (n = 73, R^2 = 0.39; P = <0.0001). Abbreviation: C_T cycle threshold

Supplementary Table 7. Table of Sequences / Accession Numbers

Sample Number	PGU Reference	GenBank Accession
Pt1_S1_E4	Adeno_2008	MW686851
Pt1_S2_E4	Adeno_2010	MW686852
Pt2_S1_C1	Adeno_2020	-
Pt3_S1_A31	Adeno_2009	MW686758
Pt4_S1	Adeno_CH_2100	Failed to sequence
Pt5_S1_C89	Adeno_2063	-
Pt5_S2_C89	Adeno_2064	-
Pt5_S3_C89	Adeno_2065	-
Pt6_S1_A31	Adeno_2023	MW686759
Pt7_S1_C1	Adeno_CH_2105	-
Pt8_S1_A31	Adeno_2024	MW686760
Pt8_S2_A31	Adeno_2022	MW686761
Pt9_S1_C5	Adeno_2043	-
Pt9_S2_C5	Adeno_2044	-
Pt9_S3_C5	Adeno_2045	-
Pt9_S4_C5	Adeno_2046	-
Pt9_S5_C5	Adeno_2048	-
Pt9_S6_C5	Adeno_2013	-
Pt9_S7_C5	Adeno_2014	-
Pt9_S8_C5	Adeno_CH_2084	-
Pt9_S9_C5	Adeno_2069	-
Pt9_S10_C5	Adeno_2070	-
Pt9_S11_C5	Adeno_2072	-
Pt9_S12_C5	Adeno_CH_2087	-
Pt9_S13_C5	Adeno_CH_2088	-
Pt9_S14_C5	Adeno_CH_2089	-
Pt9_S15_C5	Adeno_CH_2090	-
Pt9_S16_C5	Adeno_CH_2091	-
Pt10_S1_C89	Adeno_2001	-
Pt10_S2_C89	Adeno_2003	-
Pt11_S1_A31	Adeno_CH_2094	MW686762
Pt11_S2_A31	Adeno_CH_2096	MW686763
Pt12_S1_C1	Adeno_2019	-
Pt13_S1_F41	Adeno_2060	MW686853
Pt14_S1_B3	Adeno_CH_2107	MW686785
Pt15_S1	Adeno_CH_2097	Failed to sequence
Pt15_S2_C5	Adeno_CH_2101	-
Pt15_S3_C5	Adeno_CH_2102	-
Pt15_S4_C5	Adeno_CH_2103	-
Pt15_S5_C5	Adeno_CH_2104	-
Pt16_S1_C1	Adeno_CH_2074	MW686790
Pt16_S2_C1	Adeno_2049	MW686792
Pt16_S3_C1	Adeno_2051	MW686794
Pt16_S4_C1	Adeno_2052	MW686796
Pt16_S5_C1	Adeno_2005	MW686798
Pt16_S6_C1	Adeno_2053	-
Pt16_S7_C1	Adeno_2007	MW686800
Pt16_S8_C1	Adeno_2054	MW686802
Pt16_S9_C1	Adeno_2055	MW686804
Pt16_S1_C5	Adeno_CH_2074	MW686791
Pt16_S2_C5	Adeno_2049	MW686793

Pt16_S3_C5	Adeno_2051	MW686795
Pt16_S4_C5	Adeno_2052	MW686797
Pt16_S5_C5	Adeno_2005	MW686799
Pt16_S6_C5	Adeno_2053	-
Pt16_S7_C5	Adeno_2007	MW686801
Pt16_S8_C5	Adeno_2054	MW686803
Pt16_S9_C5	Adeno_2055	MW686805
Pt17_S1_C1	Adeno_2056	-
Pt18_S1_A31	Adeno_2057	-
Pt18_S2_A31	Adeno_2059	MW686757
Pt19_S1_C2	Adeno_CH_2075	-
Pt19_S2_C2	Adeno_CH_2076	-
Pt19_S3_C2	Adeno_CH_2077	-
Pt19_S4_C2	Adeno_CH_2078	-
Pt19_S5_C2	Adeno_2012	-
Pt19_S6_C2	Adeno_CH_2081	-
Pt19_S7_C2	Adeno_2026	MW686806
Pt19_S8_C2	Adeno_2027	-
Pt19_S9_C2	Adeno_2028	-
Pt19_S10_C2	Adeno_2029	MW686807
Pt19_S11_C2	Adeno_2030	MW686808
P19_S12_C2	Adeno_2031	MW686809
Pt19_S13_C2	Adeno_2032	MW686810
Pt19_S14_C2	Adeno_2033	MW686811
Pt19_S15_C2	Adeno_2034	MW686812
Pt19_S16_C2	Adeno_2035	MW686815
Pt19_S17_C2	Adeno_2036	MW686813
Pt19_S18_C2	Adeno_2037	MW686817
Pt19_S19_C2	Adeno_2038	MW686816
Pt19_S20_C2	Adeno_2039	MW686814
Pt19_S21_C2	Adeno_2040	MW686818
Pt19_S22_C2	Adeno_2041	-
Pt19_S23_C2	Adeno_2042	-
Pt19_S24_C2	Adeno_2047	MW686819
Pt19_S25_C2	Adeno_CH_2082	-
Pt19_S26_C2	Adeno_CH_2083	-
Pt19_S27_C2	Adeno_2015	-
Pt19_S28	Adeno_CH_2085	Failed to sequence
Pt19_S29_C2	Adeno_CH_2086	-
Pt20_S1_C5	Adeno_2058	-
Pt21_S1_C5	Adeno_2071	-
Pt22_S1_C1	Adeno_2002	-
Pt22_S2_C1	Adeno_CH_2073	-
Pt22_S1_C5	Adeno_2002	-
Pt22_S2_C5	Adeno_CH_2073	-
Pt23_S1_C1	Adeno_CH_2106	-
Pt23_S2_C1	Adeno_CH_2109	-
Pt24_S1_A31	Adeno_2011	MW686764
Pt24_S2_A31	Adeno_CH_2079	MW686765
Pt24_S3_A31	Adeno_CH_2080	MW686766
Pt24_S4_A31	Adeno_2025	MW686767
Pt25_S1_C1	Adeno_2050	MW686824
Pt26_S1_C1	Adeno_CH_2110	MW686825
Pt27_S1_B3	Adeno_2004	MW686786
Pt27_S2_B3	Adeno_2006	MW686787

Pt28_S1_C5	Adeno_2118	-
Pt29_S1_A31	Adeno_2062	MW686772
Pt30_S1_F41	Adeno_2061	MW686857
Pt31_S1	Adeno_2066	Failed to sequence
Pt32_S1_C2	Adeno_CH_2108	-
Pt33_S1_C5	Adeno_CH_2098	-
Pt33_S2_C5	Adeno_CH_2099	-
Pt34_S1_C89	Adeno_2111	MW686820
Pt34_S2_C89	Adeno_2114	-
Pt34_S3_C89	Adeno_2113	-
Pt34_S4_C89	Adeno_2112	-
Pt34_S5_C89	Adeno_2115	-
Pt34_S6_C89	Adeno_2116	-
Pt34_S7_C89	Adeno_2117	-
Pt35_S1_C2	Adeno_CH_2092	-
Pt35_S2_C2	Adeno_CH_2093	-
Pt35_S3_C2	Adeno_CH_2095	-
Pt36_S1_C1	Adeno_2068	MW686826
Pt37_S1	Adeno_2067	Failed to sequence
Pt38_S1_C1	CS10_1_Adenovirus	-
Pt39_S1_C1	CS41_Adeno	MW686821
Pt39_S2_C1	CS42_Adeno	MW686822
Pt39_S3_C1	CS43_Adeno	MW686823
Pt39_S4_C1	CS4_1_Adenovirus	-
Pt39_S5_C1	CS9_1_Adenovirus	-
Pt40_S1_S2_C1	CS44_Adeno	MW686827
Pt40_S3_C1	CS7_1_Adenovirus	-
Pt40_S4_C1	CS8_1_Adenovirus	-
Pt41_S1_A31	CS22_Adeno	MW686777
Pt41_S2_A31	CS20_Adeno	MW686778
Pt41_S3_A31	CS29_Adeno	MW686779
Pt41_S4_A31	CS45_Adeno	MW686780
Pt41_S5	CS34_Adeno	Failed to sequence
Pt41_S6_A31	CS51_Adeno	MW686781
Pt42_S1_C1	CS2_1_Adenovirus	MW686828
Pt42_S2_C1	CS46_Adeno	MW686831
Pt42_S3_C1	CS47_Adeno	MW686832
Pt42_S4	CS21_Adeno	Failed to sequence
Pt42_S5_C1	CS23_Adeno	-
Pt43_S1_C89	CS40_Adeno	-
Pt44_S1_C1	CS30_Adeno	MW686833
Pt44_S2_C1	CS33_Adeno	-
Pt45_S1_C2	CS37_Adeno	MW686850
Pt46_S1_A31	CS28_Adeno	MW686783
Pt47_S1_C1	CS38_Adeno	MW686834
Pt48_S1_C5	CS32_Adeno	MW686844
Pt49_S1_C1	CS36_Adeno	MW686843
Pt50_S1	CS31_Adeno	Failed to sequence
Pt51_S1_C89	CS39_Adeno	-
Pt52_S1_C2	CS35_Adeno	MW686829
Pt52_S2_C2	CS27_Adeno	MW686830
Pt52_S1_A31	CS35_Adeno	MW686770
Pt52_S2_A31	CS27_Adeno	MW686769
Pt53_S1_C5	CS55_Adeno	MW686845

Pt53_S2_C5	CS56_Adeno	MW686847
Pt53_S3_C5	CS57_Adeno	MW686846
Pt54_S1_C5	CS67_Adeno	MW686848
Pt55_S1_A31	CS66_Adeno	MW686768
Pt56_S1_C1	CS65_Adeno	MW686835
Pt57_S1_A31	CS64_Adeno	MW686771
Pt58_S1_C2	CS74_Adeno	MW686836
Pt58_S1_A31	CS74_Adeno	MW686775
Pt59_S1_B11	CS75_Adeno	MW686789
Pt60_S1_C5	CS76_Adeno	MW686849
Pt61_S1_C2	CS77_Adeno	MW686837
Pt62_S1_A31	CS78_Adeno	MW686773
Pt63_S1_F41	CS79_Adeno	MW686856
Pt64_S1_C2	CS80_Adeno	MW686840
Pt64_S1_A31	CS80_Adeno	-
Pt65_S1_F41	CS81_Adeno	MW686854
Pt66_S1_C2	CS82_Adeno	MW686841
Pt67_S1_C2	CS83_Adeno	MW686842
Pt68_S1_A31	CS84_Adeno	MW686774
Pt69_S1_A31	CS85_Adeno	MW686776
Pt69_S1_C2	CS85_Adeno	-
Pt70_S1_A31	CS86_Adeno	MW686784
Pt71_S1_F41	CS87_Adeno	MW686855
Pt72_S1_C1	CS88_Adeno	MW686839
Pt73_S1_A31	CS89_Adeno	MW686782
Pt73_S1_B3	CS89_Adeno	MW686788
Pt74_S1_C1	CS90_Adeno	MW686838

-Samples not achieving >90% average coverage
